# Identification of a novel microRNA-141-3p/Forkhead box C1/β-catenin axis associated with rheumatoid arthritis synovial fibroblast function in vivo and in vitro

**DOI:** 10.7150/thno.45214

**Published:** 2020-04-06

**Authors:** Jun Wang, Yin Wang, Hui Zhang, Jun Chang, Ming Lu, Weilu Gao, Wendong Liu, Yetian Li, Li Yin, Xiaohe Wang, Yuejun Wang, Mengru Gao, Zongsheng Yin

**Affiliations:** 1 Department of Orthopaedics, The First Affiliated Hospital of Anhui Medical University, Anhui, China; 2 Department of Plastic Surgery, The Fourth Affiliated Hospital of Anhui Medical University, Anhui, China; 3 Department of Orthopaedics, The Fourth Affiliated Hospital of Anhui Medical University, Anhui, China; 4 Department of Radiology, The First Affiliated Hospital of Anhui Medical University, Anhui, China; 5 Department of Pathology, The Fourth Affiliated Hospital of Anhui Medical University, Anhui, China

**Keywords:** rheumatoid arthritis, FoxC1, miR-141-3p, synovial fibroblasts, collagen-induced arthritis

## Abstract

**Rationale**: Rheumatoid arthritis (RA) is a prototype of inflammatory arthritis in which synovial fibroblasts (SFs) play key roles in cartilage and bone destruction through tumor-like proliferation, migration, invasion and inflammation. This study aimed to research forkhead box protein C1 (FoxC1) and microRNA (miR)-141-3p, which modulate pathological changes in the synovial membrane, to find possible strategies for treating RA.

**Methods**: FoxC1, β-catenin and miR-141-3p gene expression in synovial tissues and SFs was quantified by real-time PCR; FoxC1 and β-catenin protein levels were evaluated by immunohistochemistry, immunofluorescence, and Western blotting. We transiently transfected human SFs with FoxC1 and β-catenin overexpression and silencing vectors and assessed proliferation, migration, invasion and inflammation by cell function and enzyme-linked immunosorbent assays. We also assessed downstream signaling activation using immunofluorescence, real-time PCR and Western blotting. Double luciferase, coimmunoprecipitation and chromatin immunoprecipitation assays were used to verify miR-141-3p, FoxC1 and β-catenin gene and protein combinations. Finally, the therapeutic effects of FoxC1 silencing and miR-141-3p overexpression were evaluated in type II collagen-induced arthritis (CIA) rats.

**Results**: We found that FoxC1 expression was significantly upregulated in synovium and SFs in both RA patients and rats with collagen-induced arthritis (CIA). FoxC1 overexpression increased β-catenin messenger RNA (mRNA) and protein levels and upregulated cyclin D1, c-Myc, fibronectin and matrix metalloproteinase 3 (MMP3) mRNA and protein expression in RA SFs (RASFs). In contrast, FoxC1 knockdown reduced β-catenin mRNA and protein levels as well as cyclin D1, c-Myc, and fibronectin mRNA and protein levels in RASFs. Furthermore, altering FoxC1 expression did not significantly change GSK3β and pGSK3β levels. FoxC1 overexpression promoted proliferation, migration, invasion and proinflammatory cytokine (interleukin (IL)-1β, IL-6, and tumor necrosis factor-α (TNF-α)) production and reduced anti-inflammatory cytokine (IL-10) levels in RASFs. FoxC1 bound to the β-catenin promoter, and β-catenin mediated the FoxC1-induced pathological changes. We also observed downregulated microRNA (miR)-141-3p expression in SFs from both RA patients and CIA rats and further found that miR-141-3p bound to the FoxC1 3′UTR and suppressed FoxC1 expression. Intra-ankle miR-141-3p agomir or FoxC1-specific siRNA injection hindered CIA development in rats.

**Conclusions**: FoxC1 and miR-141-3p participate in RA pathogenesis by mediating inflammation and SF proliferation, migration, and invasion and thus could be novel targets for RA therapy as a nonimmunosuppressive approach.

## Introduction

Rheumatoid arthritis (RA), a prototype disease for the study of inflammatory arthritis, is characterized by increased synovial inflammation and progressive cartilage and bone resorption, which lead to chronic inflammation, poor joint health and unfavorable prognoses 1. Research on RA has resulted in immunotherapies targeting inflammatory and immune signaling pathways and pathogenic components, and while these treatments are effective, few patients with RA are able to maintain remission without drugs 2. Innovative treatment options are needed to gain new insights into the pathogenesis of the disease and identify potential new treatments.

Synovial inflammation is caused by infiltration of innate and adaptive immune cells, including activated resident synovial fibroblasts (SFs), and is the major pathological change of RA 3-5. The main role of SFs is to provide synovial tissue structural support, secrete synovial fluid to lubricate joints, reduce frictional movements, and nourish avascular cartilage 6, 7. However, in the synovial membrane of RA, SFs are activated and show the characteristics of reduced apoptosis, migration, and invasion, becoming the main effector cells of invasive pannus and actively participating in the inflammatory process of RA 8, 9. These activated SFs exhibit an aggressive phenotype with a tumor-like appearance and play major roles in RA by producing proinflammatory cytokines such as IL-1β 10, TNF-α 11 and IL-6 12, 13 as well as matrix metalloproteinases and angiogenic factors 14. Similar to other types of cells with malignant tumor phenotypes, RASFs have an inherent capability to resist a repellent synovial environment, which is rich in oxygen free radicals and other toxic metabolites under hypoxic conditions 15, 16. Thus, changes in the RASF phenotype, which promotes the formation of synovial pannus and invades adjacent cartilage and bone, are key to RA formation. The currently available drugs for RA may not directly target the disordered RASF phenotype, resulting in a lack of treatments for patients with this phenotype. The specific activation mechanism of RASFs is still unknown, but recent studies have found that the Wnt/β-catenin pathway may be involved in the activation of RASFs and RA pathogenesis 17, 18. The Wnt/β-catenin pathway plays an important role in the regulation of cell growth, proliferation, migration, differentiation, self-renewal, homeostasis, and embryonic development, as well as the occurrence and development of various malignant tumors 19, 20. In the normal physiological function of tissues and organs, Wnt/β-catenin is usually inactivate. However, when tissues and organs are damaged, Wnt/β-catenin is often activated 21, 22. β-catenin expression is widely regarded as a sentinel marker under pathological conditions of the Wnt/β-catenin pathway 23, 24.

The members of the Forkhead box (Fox) transcription factor family share a 100-amino acid winged helix DNA-binding domain that plays an important role in metabolism, differentiation, proliferation, apoptosis, migration, invasion and longevity in cells 25. Recent studies have shown that the Fox family member FoxC1 participates in mesoderm, brain and eye development during embryogenesis 26, 27 and may be important in cancer pathology 28, 29. Researchers have found that NF-κB signaling mediates the function of FoxC1 during basal-like breast cancer (BLBC) cell proliferation and invasion 30. IL-8 activates FoxC1 expression via the PI3K/AKT pathway and via hypoxia-inducible factor 1 alpha, and FoxC1 expression induces CXCR1 and CCL2 transactivation and promotes inflammation in hepatocellular carcinoma (HCC) and the migration and invasion of HCC cells 31. FoxC1 also promotes proliferation, migration, invasion and drug resistance in HCC 32 and lung cancer 33. These findings highlight the role of FoxC1 in tumor formation and progression; however, the role of FoxC1 in RASFs remains unclear.

MicroRNAs (miRNAs or miRs) are short (~22 nucleotides long) noncoding RNAs that, as post-transcriptional regulators, play a key role in a variety of cellular functions. MiRNAs primarily silence target genes by binding to the 3' untranslated region (3′UTR) or 5' untranslated region (5′UTR) of messenger RNA (mRNA) and specifically inhibiting mRNA translation or inducing mRNA degradation 34. Thus far, numerous studies have shown that miRNA dysfunction is associated with inflammatory and autoimmune diseases 35. For example, research has revealed that miR-155 36, miR-146a 37, miR-20a 38, miR-124 39 and miR-223 40 are differentially expressed in arthritis models, suggesting the importance of their roles in balancing immune activation and RA pathogenesis 41. Therefore, miRNAs have attracted extensive attention as potential therapeutic targets, and their sequence-specific patterns enable the simultaneous targeting of multiple genes 42.

In this study, we explored the effects of FoxC1 on tumor-like properties and inflammatory responses of RASFs and the potential underlying mechanisms. We found that RASFs exhibited strong proliferation and migration capabilities that were decreased by FoxC1 knockdown. In addition, upon FoxC1 silencing, proinflammatory factors (IL-1β, IL-6, and TNF-α) were downregulated, while an anti-inflammatory factor (IL-10) was upregulated. FoxC1 interacted directly with β-catenin to activate canonical Wnt signaling, and regulating the expression of β-catenin reverses a series of pathological changes caused by FoxC1. Furthermore, miR -141-3p was found to bind to the FoxC1 3'UTR and mediates FoxC1 and β-catenin expression. These results show that the miR-141-3p/FoxC1/β-catenin pathway is associated with RA pathogenesis and may provide new biomarkers and therapeutic targets for RA research and treatment.

## Materials and methods

### Acquisition of synovial specimens of joints

Normal synovium tissues were obtained from amputees (n=4) or patients requiring arthroscopic knee surgery (n=6). Patients with other musculoskeletal disorders were excluded. RA-associated human articular synovium samples were obtained from patients (n=20) who fulfilled the American College of Rheumatology criteria for the classification of RA 43 and who were undergoing total knee joint arthroplasty surgery. The study was conducted under the guidance of the Helsinki and Tokyo Declarations of Human Rights. All human studies were conducted upon receiving informed consent from the patients and approved by the institutional ethics review committee of the First Affiliated Hospital of Anhui Medical University. The basic information for the patients is shown in **Table S1**.

### Cell isolation and culture

SFs were isolated from synovial tissues of patients as previously described 44. To ensure good biological function of the SFs, primary cells were used after 3-5 passages. To confirm the identity of isolated SFs, antibodies targeting vimentin (BD Bioscience) and CD68 (BD Bioscience) were used to detect the expression levels via flow cytometry. Normal SFs (2×10^5^ cells) were stimulated with 1 mL of medium alone or with medium containing lipopolysaccharide (LPS; 1 μg/mL).

### Histological, immunohistochemical and immunofluorescence analyses

Synovial tissues were fixed in 4% paraformaldehyde (Beyotime, Shanghai, China) at 4 °C and embedded in paraffin. Six-micrometer-thick tissue sections were stained with hematoxylin and eosin (HE). The paraffin-embedded sections were processed, further stained with anti-FoxC1 antibodies (ab223850, Abcam, Cambridge, UK), β-catenin (D10A8) XP® rabbit mAbs #8480 (Cell Signaling, Beverly, MA, USA), anti-IL-1β antibodies (ab9722) (Abcam), anti-IL-6 antibodies (ab9324) (Abcam), anti-TNF-α antibodies (ab6671) or anti-IL-10 antibodies (ab192271) (Abcam), and then incubated with secondary antibodies (ZSGB-BIO, Beijing, China) and immunohistochemical staining substrates, as previously described 45. Imaging was performed using an Olympus IX81 imaging system. Staining intensity was semiquantitated using the H-score method as described previously 46-48. Two pathologists who were blinded to the clinicopathological information examined and scored all the immunostained sections. Ten randomly selected fields of view at a magnification of 400× were observed. The staining intensity was scored as follows 46-48: 0, none; 1+, weak; 2+, medium; and 3+, strong. The total number of cells and the number of stained cells at each intensity were counted for each field of view. The H-score was calculated according to the following formula: (Percentage of cells stained at intensity classification 1 × 1) + (Percentage of cells stained at intensity classification 2 × 2) + (Percentage of cells stained at intensity classification 3 × 3). H-scores range from 0 to 300, with 300 indicative of 100% of cells with strong staining (3+). High protein expression was defined as H-scores of ≥200.

For immunofluorescence analysis, SFs were plated at a density of 10,000 cells/well in a 12-well cell culture plate (Corning Incorporated, Corning, NY, USA) with coverslips. After treatment, the cells were fixed with 4% paraformaldehyde (Beyotime) at room temperature for 20 min and incubated with anti-FoxC1 antibodies (ab223850) (Abcam, Cambridge, UK) or β-catenin (D10A8) XP® rabbit mAbs #8480 (Cell Signaling, Beverly, MA, USA) at 4 °C overnight. The cells were then incubated with a FITC-conjugated secondary antibody (ZSGB-BIO, Beijing, China) for 1 h and with DAPI (Beyotime) to stain the nuclei. The fluorescence was observed under a fluorescence microscope (Olympus IX81, Japan).

### Quantitative real-time PCR (qRT-PCR)

Total RNA was extracted from synovial tissues or SFs with 1000 μL of TRIzol Reagent (Invitrogen, USA) and then treated with DNase (Thermo Scientific). Next, the RNA concentration was quantified on a NanoDrop One (Thermo Scientific), and equal amounts of total RNA (500 ng) were reverse-transcribed to synthesize cDNA in a total reaction volume of 10 μL per sample using a PrimeScript^TM^ RT Reagent Kit (Takara, Dalian, China). Then, qRT-PCR analyses of the target genes were performed using a SYBR® Premix Ex Taq^TM^ II Kit (Takara, Dalian, China) in 20 μL reactions containing SYBR (10 μL), primers (0.8 μL each of the forward and reverse primers), cDNA template (2.0 μL) and ddH2O (6.0 μL). The PCR program included an activation step at 95 °C for 30 s followed by 40 cycles of 5 s at 95 °C and 34 s at 60 °C. Target mRNA expression was normalized to β-actin expression in each sample and compared to the control sample.

MiRNA reverse transcription was performed using a TaqMan MicroRNA RT kit (Life Technologies), and qRT-PCR was performed using TaqMan Universal Master Mix (Life Technologies) according to the kit instructions. Each 15 μL RT reaction contained 10 ng of total RNA (5 μL), 0.15 μL of each dNTP (100 mM total), 1.0 μL of MultiScribe RT (50 U/μL), 50 nM stem-loop reverse transcriptase primers (3.0 μL each), 0.19 μL of RNase inhibitor (20 U/μL), 1.5 μL of 10×RT buffer and 4.16 μL of nuclease-free water; the reactions were incubated for 30 min at 16 °C, 30 min at 42 °C, and 5 min at 85 °C. For qRT-PCR, 10.0 μL of TaqMan Universal PCR Master Mix, 1.33 μL of cDNA, 1.0 μL of primer and 7.67 μL of nuclease-free water were combined in 20 μL reactions, and the reaction program consisted of a single step of 10 min at 95 °C and 40 cycles of 15 s at 95 °C and 60 s at 60 °C.

The relevant primer sequences are shown in **Table S2**. The relative quantity for each experiment was calculated automatically using the comparative quantitation mode of MxPro qRT-PCR system software. The relative mRNA expression was calculated using the 2-^ΔΔCt^ method. At least three independent experiments were performed and analyzed.

### Western blotting

Synovium and SFs were lysed using RIPA buffer (Beyotime, China) supplemented with a protease inhibitor cocktail (Beyotime), and the extraction of SF nuclear proteins was performed according to the method of the nuclear protein extraction kit (Beyotime). The total and nuclear protein concentrations were determined using a BCA protein assay kit (Beyotime) according to the manufacturer's instructions. Thirty micrograms of protein per sample was separated by SDS-PAGE, and the separated proteins were transferred onto 0.45 μm PVDF membranes (Immobilon^TM^, Millipore Corp, Bedford, MA). After the membrane was sealed with milk, the corresponding protein bands were cut out according to the marker and then incubated overnight at 4 °C in the corresponding primary antibodies as follows: anti-FoxC1 (ab223850, Abcam, Cambridge, UK); anti-FoxC1 (ChIP grade, ab5079, Abcam); anti-fibronectin (ab45688, Abcam); anti-MMP3 (ab52915, Abcam) and anti-histone H3 (ab61251, Abcam); anti-β-catenin (D10A8, Cell Signaling, Beverly, MA, USA); anti-GSK-3α/β (D75D3, Cell Signaling); anti-c-Myc (D84C12, Cell Signaling); anti-cyclin D1 (#2922, Cell Signaling); anti-β-actin (8H10D10, Cell Signaling) and anti-GAPDH (D16H11, Cell Signaling). The antibodies were detected using HRP-conjugated secondary antibodies anti-mouse IgG (#7076, Cell Signaling) or anti-rabbit IgG (#7074, Cell Signaling). A super-sensitive chemiluminescence (ECL) kit (Tanon, Shanghai, China) was used to detect chemiluminescence signals. We used ImageJ software (National Institutes of Health, USA) to quantify the gray values.

### SiRNA and miRNA transfection

Small interfering RNAs (siFoxC1 and siβ-catenin) were used to inhibit the expression of FoxC1 and β-catenin. miR-141-3p mimic and miR-141-3p inhibitor were used to overexpress and inhibit miR-141-3p. All of these compounds and the corresponding negative controls (negative control siRNA, negative control miRNA) were purchased from RiboBio (Guangzhou, China). According to the manufacturer's protocol (RiboBio), the siRNAs, miRNA mimic, miRNA inhibitor and negative controls were transfected into SFs using the riboFECT™ CP Transfection Kit (RiboBio) at an effective concentration of 50 nM. The siRNA and miRNA transfection efficiencies were monitored by qRT-PCR and Western blot analysis at 48-72 h after transfection. The sequences of siFoxC1, siβ-catenin, miR-141-3p mimic and miR-141-3p inhibitor are shown in **Table S3**.

### Transfection of lentiviral vectors that overexpress FoxC1 or β-catenin

Lentiviral vectors that overexpress full-length human FoxC1 (LVFoxC1) or β-catenin (LVβ-catenin), and corresponding negative control (LVcontrol), were purchased from Hanbio (Shanghai, China). The lentivirus was transfected with SFs at the concentration recommended by Hanbio (MOI=1:20) and with the corresponding transfection enhancer polybrene according to the manufacturer's protocol. The cells were used for additional assays 72 h after transfection.

### Cell proliferation, migration and invasion assays

To assess proliferation, SFs in each group at 48-72 h after transfection were collected, counted and adjusted to a density of 5×10^4^ cells per 100 μL. Then, the SFs were seeded in a 96-well plate, and subsequent experiments were carried out at four time points: 0 h, 24 h, 48 h, and 72 h. A total of 10 μL of Cell Counting Kit-8 (CCK-8, Beyotime) reagent was added to each well at the corresponding time point, and the cells were cultured at 37°C for 2 h before the absorbance of each well was measured at a wavelength of 450 nm on a Thermomax microplate reader (Bio-Tek-El, USA).

To assess migration, SFs in each group at 48-72 h after transfection were cultured in a 6‑well plate in high-glucose DMEM (HyClone, South Logan, Utah, USA) containing 10% FBS (CLARK, Richmond, VA, USA) and incubated at 37 °C (initial cell density 1×10^5^ cells/well) to 100% confluence. Next, a wound was created in the cell monolayer with a plastic scriber. The cells were then washed and again cultured in high-glucose DMEM with 10% FBS at 37 °C for 48 h. The wounds were photographed under a microscope (Olympus IX81), and the wound area was evaluated with ImageJ software.

Invasion assays were conducted using transwell chambers with 8-μm pores (Corning Incorporated). After transfection for 48-72 h, 2.5×10^5^ cells in 200 μL of high-glucose DMEM were seeded in the upper chamber of membranes precoated with Matrigel (BD Bioscience). Next, 800 μL of high-glucose DMEM with 10% FBS was added to the lower chamber. After the cells were incubated at 37 °C for 48 h, those on the upper surface were gently wiped away, whereas invading cells present on the lower membrane were fixed with 4% paraformaldehyde and stained with a 0.1% crystal violet solution. Finally, we counted five randomly selected high power fields per well to assess the average number of invading cells.

### Enzyme-linked immunosorbent assay (ELISA)

The levels of the cytokines IL-1β, IL-6, TNF-α and IL-10 in the supernatants obtained from SF cultures were measured using corresponding ELISA kits (ab46052, ab46027, ab181421, and ab46034, respectively; Abcam) according to the manufacturer's protocol. The optical density values at 450 nm were recorded.

### Luciferase reporter assay

To confirm that FoxC1 expression was regulated by binding of miR-141-3p to the FoxC1 3′UTR, we used a pSI-Check2 reporter vector containing the miR-binding sequences of the FoxC1 3′UTR (Hanbio) in combination with a Dual-Luciferase Reporter Assay System (Promega, USA). RASFs were co-transfected with plasmids carrying miR-141-3p pre-miRNA (premiR-141-3p), negative control pre-miRNA (premiR-NC), wild-type 3'UTR of FoxC1 (3'UTR wt) and the 3'UTR mutation of FoxC1 (3'UTR mut). Lipofectamine 3000 (Invitrogen) was used for plasmid transfection according to the manufacturer's instructions. After 48 h, the Dual-Luciferase Reporter Assay System was employed to measure the luciferase activity. Firefly luciferase activity was normalized to the corresponding Renilla luciferase activity.

The full-length FoxC1 cDNA was cloned into the pcDNA3.1 vector (Hanbio) to construct the FoxC1-expressing plasmid pcDNA3.1-FoxC1. Wild-type or mutant regions 2000 bp upstream of the β-catenin promoter (identified by JASPAR) (BS1, BS2, BS3, and BS4) were cloned into the pGL3 plasmid (Promega) to construct pGL3-β-catenin reporter plasmids. RASFs were transfected with pcDNA3.1-FoxC1, pGL3-β-catenin and Renilla luciferase reporter PRL-TK plasmids using Lipofectamine 3000. After 48 h, the Dual-Luciferase Reporter Assay System was employed to measure luciferase activity, and the transfection efficiencies were normalized to the Renilla activity. The dual-luciferase activity of the transfected cells was measured on a Thermomax microplate reader.

### Coimmunoprecipitation (CoIP)

RASFs were lysed in RIPA at 4 °C for 30 min, the lysates were centrifuged at 14,000 × *g* for 15 min, and the supernatants were collected. A small amount of whole-cell lysate was retained as the input. Next, the supernatant samples were incubated with the FoxC1 antibody (ab5079), β-catenin antibody (L87A12) (Cell Signaling) or normal IgG antibody (Cell Signaling) combined with protein A/G magnetic beads (Thermo Scientific) on a rotating device at 4 °C overnight. The bead-complexes were collected by centrifugation at 14,000 × *g* for 1 min at 4 °C. Finally, the beads were washed with lysate, and the protein was boiled with 10% SDS and subjected to immunoblot analyses.

### Chromatin immunoprecipitation (ChIP)

SFs (1×10^7^ cells) were crosslinked with 1% formaldehyde (Beyotime) at 37 °C for 10 min. After the cells were washed with PBS, they were resuspended in 300 μL of lysis buffer (1% SDS, 1 mM PMSF, 50 mM Tris (pH 8.1) and 10 mM EDTA). The DNA was sheared to lengths between 200 bp and 1000 bp by sonication. The protein-DNA complexes were precipitated with a ChIP-grade-FoxC1 antibody, with normal IgG antibody serving as a negative control and anti-RNA pol-II antibody (Cell Signaling) serving as a positive control, overnight at 4 °C. Protein A/G magnetic beads were used to purify the complexes, and the cross-linkages were reversed at 68 °C for 6 h. Next, the DNA was purified using a PCR Purification Kit (Qiagen, USA). FoxC1 and RNA polymerase II protein levels in the ChIP assay products were detected by Western blotting. Finally, the binding capacity between FoxC1 and the β-Catenin promoter was detected by qRT-PCR. The β-catenin promoter primer sequences used in the ChIP-qRT-PCR assay were as follows: 5'-TTGTTTACGGTGTCAGTAGGGATTA-3' (sense) and 5'-CTGCACCATTAGAAGATCTAAAGGA-3' (antisense).

### Animal model induction and treatment

Thirty-six specific pathogen-free (SPF) female Lewis rats (weight 180-220g) were obtained from Zhejiang Vital River Laboratory (Zhejiang, China), maintained at 21 °C under a 12 h light/dark cycle and given a standard rodent diet and filtered water ad libitum. The animal experiments in this study were carried out according to the protocols approved by the Anhui Medical University Animal Care and Use Committee (No. LLSC20190547).

Collagen-induced arthritis (CIA) was established by administering bovine type II collagen emulsified in incomplete Freund's adjuvant at the base of the tail (0.1 mL) and at two sites on the back (0.2 mL for each site) on day 0 and day 7 according to previously described methods 49. The rats were divided randomly into six groups (6 rats per group): group 1 (G1), no treatment (i.e., normal controls); group 2 (G2), CIA model controls (no other treatments); group 3 (G3), model rats administered siFoxC1 (2' O-methyl (OMe)+5' cholesterol (chol)+5' Cy5-modified); group 4 (G4), model rats administered siControl (2' OMe+5' chol-modified) (RiboBio); group 5 (G5), model rats administered miR-141-3p agomir (2' OMe+5' chol+5' Cy5-modified); and group 6 (G6), model rats administered negative control agomir (2' OMe+5' chol-modified) (RiboBio). The treatments in rats in groups G3-G6 were administered via intra-articular injection (5 nmol in 0.9% saline; 30 µL volume) into their left hind ankle joints once a week for 3 weeks (**Supplementary movie**). All rats in the experimental groups were sacrificed on day 35 after the first collagen immunization. Synovial tissues from the hind knee joints (G1 and G2) were assessed by HE staining and immunohistochemistry, and rat SFs were isolated and cultured according to the method described above. Four-percent paraformaldehyde solution (Beyotime) was used to fix all of the left hind ankle joints. Micro-CT was used to scan all of the left hind ankle joints. Inflammation of synovial tissue and destruction of articular cartilage were evaluated by HE staining and Safranin O/Fast Green staining according to previous standards 50, 51.

### Joint swelling measurements and arthritis clinical scores

For the CIA model, the swelling and clinical scores of the joints were evaluated daily from the onset of arthritis (day 14) until the animals were sacrificed. Joint swelling in each rat was evaluated as the average volume of the left hind paw as measured with a plethysmometer (PV-200, Techman, China). The clinical arthritis scores for the left hind paws of the rats were obtained following a standard evaluation process 52 in which a score of 0 indicated no evidence of erythema or swelling, a score of 1 indicated erythema and mild swelling, a score of 2 indicated erythema and mild swelling extending from the ankle to the tarsals, a score of 3 indicated erythema and moderate swelling extending from the ankle to the metatarsal joints, and a score of 4 indicated erythema and either severe swelling at the ankle, foot and digits or ankylosis of the limb. The average clinical scores of the left hind paws were then calculated.

### Vivo imaging system

To understand the duration of siFoxC1 and the miR-141-3p agomir in the articular cavities of the rats, the left hind paws were imaged on day 0 and day 7 using a Vivo imaging system (PerkinElmer, USA).

### Micro-CT assessment

To evaluate bone damage, the left ankle joints of rats sacrificed at day 35 were fixed in 4% paraformaldehyde solution (Beyotime) and scanned in an ex vivo micro-CT machine (Skyscan1174 X-ray Micro-CT) (Bruker) for 160 min at 50 kV and 800 μA with a resolution of 14.5 μm. The dataset was then reconstructed using N-Reconn and CTvox software to obtain three-dimensional images of the joints and to measure bone mineral density (BMD) and other morphometric parameters. The left calcaneus in the rats was assessed for bone destruction, and its BMD was measured as a comparative indicator of bone damage. The highlighted ROI in the left calcaneus was analyzed for the following morphometric parameters 53: (1) bone volume/total tissue volume (BV/TV), (2) trabecular number (Tb.N), (3) trabecular thickness (Tb.Th), (4) trabecular separation (Tb.Sp), and (5) trabecular mesh factor (Tb.Pf).

### Bioinformatics target prediction

MiRNAs that may bind to the 3′UTR of FoxC1 were identified using the miRanda, miRWalk, miRDB, PITA, Microt4, RNA22, miRMap, RNAhybrid and TargetScan 6.0 databases. Conserved miRNAs were selected for further validation if they were predicted by all 9 of the databases mentioned above. The JASPAR database was used to predict the binding of FoxC1 with the β-catenin promoter.

### Statistical analysis

A student's unpaired 2-tailed t-test was used to analyze differences between two groups. One-way analysis of variance (ANOVA) with an LSD posttest was used for multiple comparisons. All experiments were performed in triplicate. A two-tailed P value <0.05 was considered to indicate statistical significance. All analyses were performed using IBM SPSS Statistics 22 and GraphPad Prism 7.

## Results

### FoxC1 and β-catenin are significantly upregulated in the synovium and SFs of RA patients and CIA rats

**Figure 1:**
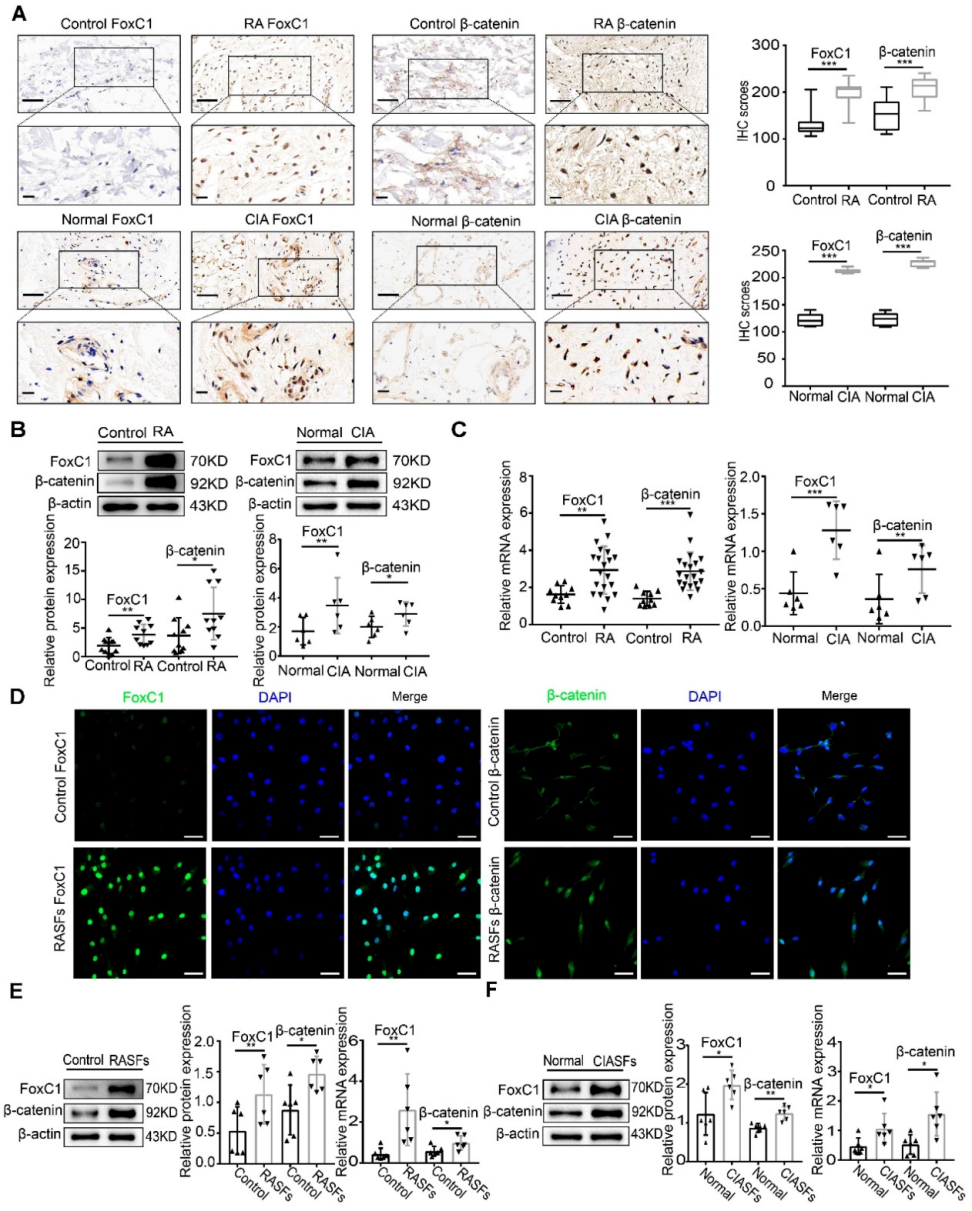
** FoxC1 and β-catenin are significantly upregulated in the synovium and SFs of RA patients and CIA rats.** (A) The expression of FoxC1 and β-catenin in synovial tissues of RA patients (n=20) and CIA rats (n=6) was detected by immunohistochemistry, and corresponding control groups (control patients (n=10), normal rats (n=6)) were set up. Typical images and IHC scores were shown. Original magnification ×200, original magnification ×400. (B) The protein expression of FoxC1 and β-catenin in synovial tissues of RA patients (n=20) and CIA rats (n=6) was detected by WB, and corresponding control groups (control patients (n=10), normal rats (n=6)) were set up. (C) The mRNA expression of FoxC1 and β-catenin in synovial tissues of RA patients (n=20) and CIA rats (n=6) was detected by RT-PCR, and corresponding control groups (control patients (n=10), normal rats (n=6)) were set up. (D) The expression sites of FoxC1 and β-catenin in RASFs were observed by immunofluorescence and the corresponding control SFs (n=4) were set up. Original magnification ×200. (E) The protein and mRNA expression of FoxC1 and β-catenin in SFs of RA patients (n=6) were analyzed by western blotting and qRT-PCR and the corresponding control SFs (n=6) were set up. (F) The protein and mRNA expression of FoxC1 and β-catenin in SFs of CIA rats (n=6) were analyzed by western blotting and qRT-PCR and the corresponding normal SFs (n=6) were set up. Experiments were independently repeated three times. The data were expressed as mean ± SD. *p<0.05, **p<0.01, ***p<0.001, t-test. Scale bars: 50 μm.

To clarify the role of FoxC1 and β-catenin in the pathogenesis of RA, we obtained synovial tissue samples from patients with RA (n=20), individuals with normal synovium (n=10), normal rats (n=6) and CIA model rats (n=6). HE staining was performed to confirm that the tissue was synovial tissue (**Figure S1A-B**). We analyzed the position and expression level of FoxC1 and β-catenin in these synovial tissue samples by IHC. FoxC1 protein levels were increased (H-score≥200) in 13 (65%) of 20 RA synovium tissues and in 4 (66.7%) of 6 CIA synovium tissues. The β-catenin protein levels were also increased (H-score≥200) in 14 (70%) of 20 RA synovium tissues and in 5 (83.3%) of 6 CIA synovium tissues. As shown in **Figure 1A**, we found that the expression of FoxC1 and β-catenin was higher in RA patients and CIA rats than in the control group, and positive staining was localized mainly in the cytoplasm and nucleus. In contrast, staining for FoxC1 and β-catenin was negative or weak in the corresponding control groups (**Figure 1A**). In addition, the FoxC1 and β-catenin staining scores were significantly higher in the RA patient and CIA rat synovium tissues than in the respective control tissues (**Figure 1A**). Western blotting and qRT-PCR results also showed that both FoxC1 and β-catenin protein and mRNA levels were higher in the RA patients and CIA rat groups compared to the respective control groups (**Figure 1B-C**).

To verify the successful isolation of RASFs, the expression levels of vimentin and CD68 were detected by flow cytometry, and the results suggested that the proportion of third-generation RASFs was very high (**Figure S1C**). qRT-PCR showed that FoxC1 mRNA expression continuously decreased in successive RASF generations. Thus, we used primary cultured SFs at passages 3-5 (**Figure S1D**). We observed the distribution of FoxC1 and β-catenin in SFs by immunofluorescence and found that FoxC1 and β-catenin were expressed in both the nucleus and cytoplasm, but mainly in the nucleus (**Figure 1D**). Immunofluorescence also revealed a greater degree of cytoplasmic and nuclear localization of FoxC1 and β-catenin in RASFs compared to that in control cells (**Figure 1D**). In addition, we found that FoxC1 was barely expressed in the cytoplasm in the control group (**Figure 1D**). Western blotting further revealed that FoxC1 and β-catenin expression levels were significantly higher in RASFs and CIA SFs (CIASFs) than in the respective control cells (**Figure 1E-F**). qRT-PCR also demonstrated that mRNA levels of FoxC1 and β-catenin were higher in RASFs and CIA SFs than in the respective control cells (**Figure 1E-F**).

These results suggest that the abnormal expression (overexpression) of FoxC1 and β-catenin may be related to the pathogenesis of RA in the synovium.

### In RASFs, FoxC1 promotes proliferation, migration, and invasion of the cells, stimulates proinflammatory cytokine production and reduces anti-inflammatory cytokine levels

**Figure 2:**
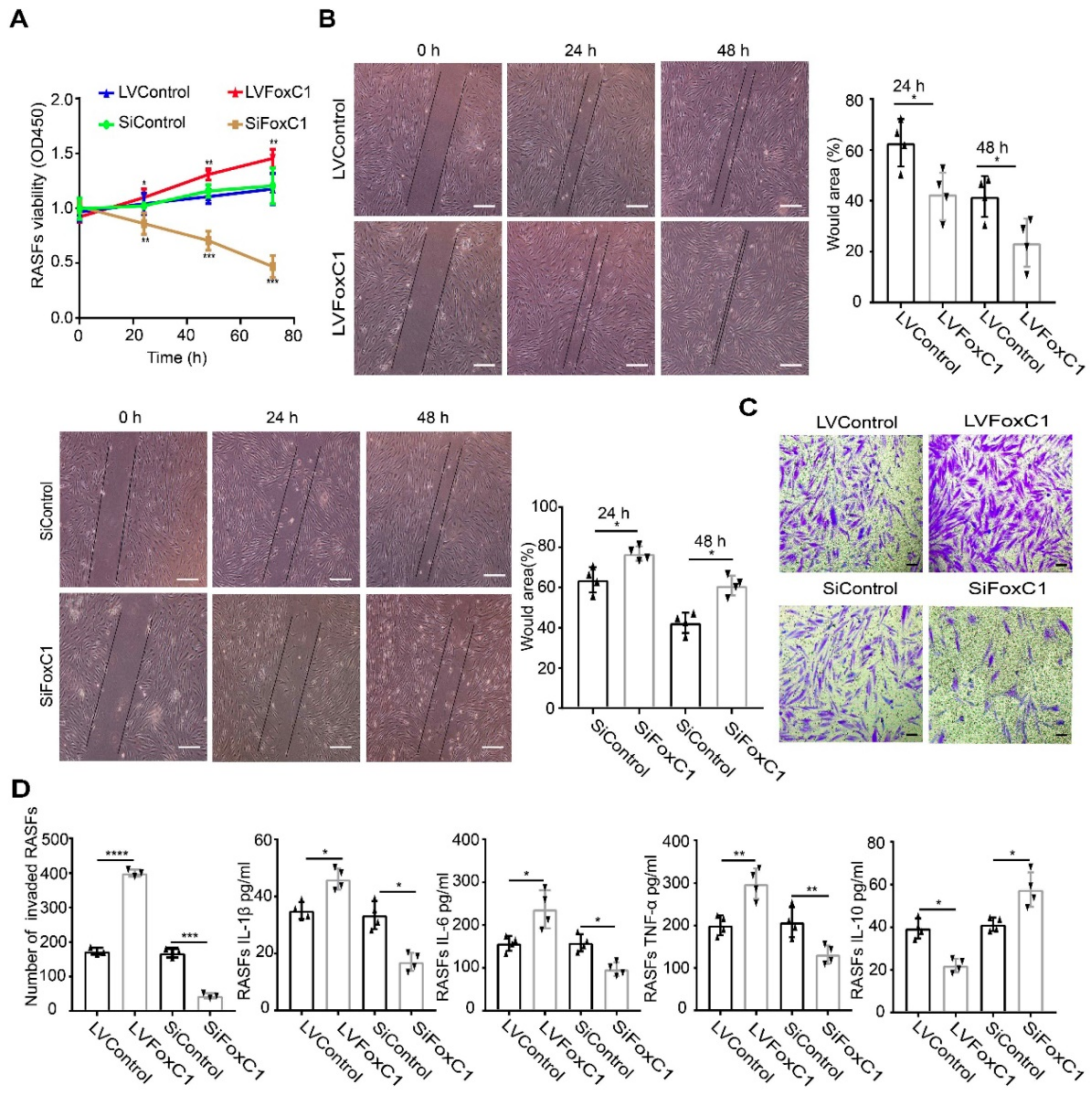
** In RASFs, FoxC1 promotes proliferation, migration, and invasion of the cells, stimulates proinflammatory cytokine production and reduces anti-inflammatory cytokine levels.** (A) The CCK-8 assay was used to evaluate cell proliferation after overexpression (LVFoxC1, n=4) or inhibition (SiFoxC1, n=4) of FoxC1 in RASFs, and corresponding control groups (n=8) were set up. (B) Wound healing through RASFs (LVFoxC1 (n=4), SiFoxC1 (n=4) and control (n=8)) migration was observed sequentially (0 h, 24 h and 48 h) by microscopy. Original magnification ×100. The black lines were used to mark the general area of the wound. Wound area was quantified by Image J. (C) Invasion of RASFs (LVFoxC1 (n=3), SiFoxC1 (n=3) and control (n=6)) was determined by transwell assays (48 h). Original magnification ×200. (D) Inflammatory cytokines concentration in culture supernatant of RASFs (LVFoxC1 (n=4), SiFoxC1 (n=4) and control (n=8)) was measured by ELISA. Experiments were independently repeated three times. The data were expressed as mean ± SD. *p<0.05, **p<0.01, ***p<0.001, ****p<0.0001, t-test. Scale bars: 100 μm.

To investigate the role of FoxC1 in RASFs, LVFoxC1 and SiFoxC1 were transfected into cells to construct RASFs that overexpressed and knocked down FoxC1, respectively. First, we evaluated cell proliferation by the CCK-8 assay. We found that the proliferation of RASFs increased after LVFoxC1 transfection (**Figure 2A**). In contrast, the proliferation of RASFs was reduced after SiFoxC1 transfection (**Figure 2A**). Next, we assessed the migration and invasion of LVFoxC1- and SiFoxC1-transfected RASFs with wound healing and transwell assays, respectively. LVFoxC1-transfected RASFs exhibited better migration and invasion than did control cells (**Figure 2B-C**), while SiFoxC1-transfected RASFs exhibited poorer migration and invasion than did control cells (**Figure 2B-C**). We also determined whether FoxC1 overexpression affected the production of RA-related inflammatory cytokines in RASFs. ELISA results confirmed that after LVFoxC1 transfection in RASFs, the expression levels of IL-1β, IL-6, and TNF-α were higher and the expression levels of IL-10 were lower in the cell supernatants than in the control group; in contrast, the levels of IL-1β, IL-6, and TNF-α were lower and the levels of IL-10 were higher in the cell supernatants from SiFoxC1-transfected RASFs than those from the control group (**Figure 2D**). These data may indicate that FoxC1 is involved in a series of pathological changes of SFs in RA by inducing changes in SF proliferation, migration, invasion, and production of inflammatory cytokines.

### FoxC1 induces pathological changes in RASFs through β-catenin

**Figure 3:**
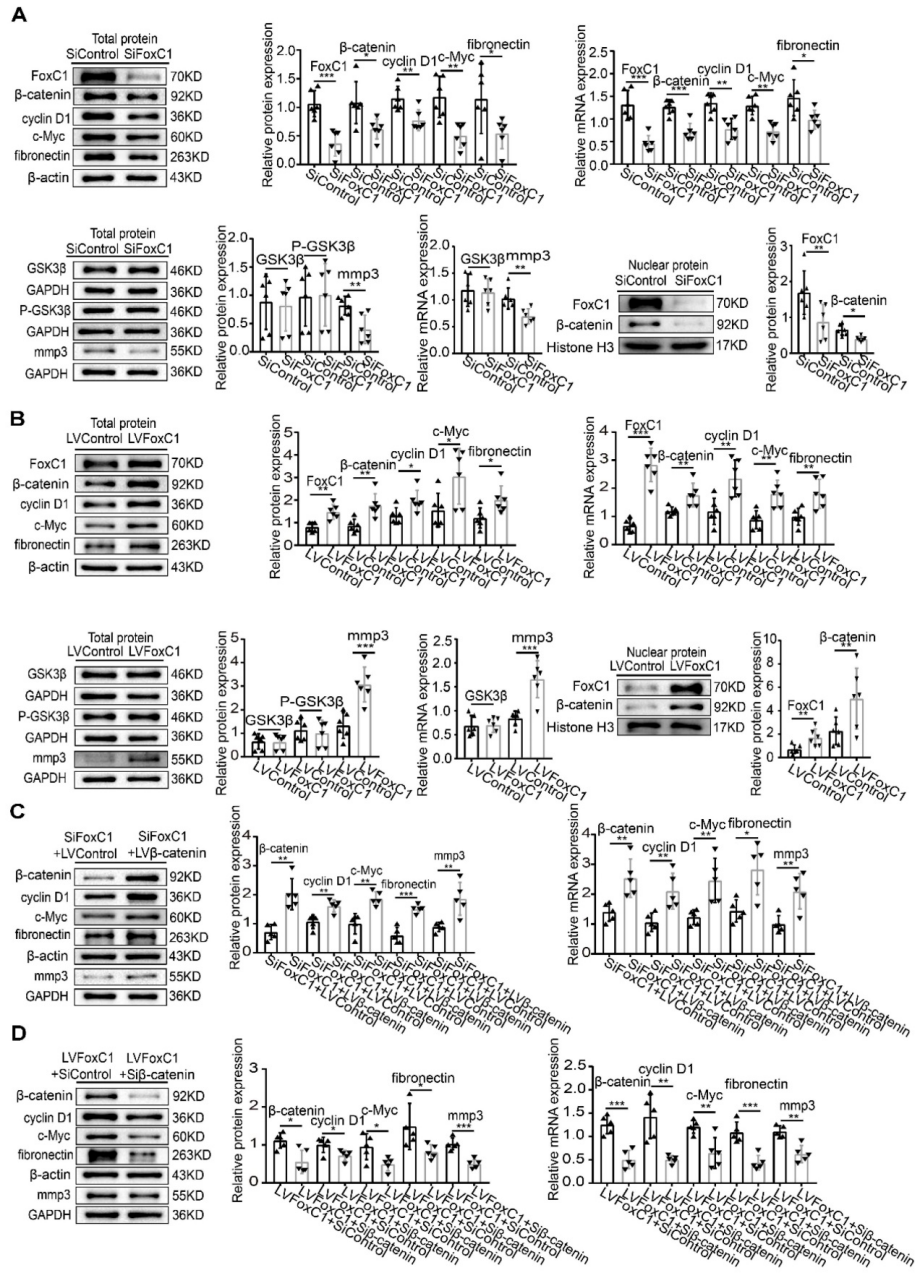
** FoxC1 induces pathological changes in RASFs through β-Catenin.** (A) WB and qRT-PCR methods were used to detect protein and gene changes in FoxC1, β-catenin, GSK3β, cyclin D1, c-Myc, fibronectin, and MMP3 in FoxC1 inhibited RASFs (SiFoxC1, n=6), and corresponding control group (n=6) was set. (B) WB and qRT-PCR methods were used to detect protein and gene changes in FoxC1, β-catenin, GSK3β, cyclin D1, c-Myc, fibronectin, and MMP3 in FoxC1 overexpressed RASFs (LVFoxC1, n=6), and corresponding control group (n=6) was set. (C) WB and qRT-PCR methods were used to detect protein and gene changes in β-catenin, cyclin D1, c-Myc, fibronectin, and MMP3 in overexpression β-catenin SiFoxC1-RASFs (n=5), and corresponding control group (n=5) was set. (D) WB and qRT-PCR methods were used to detect protein and gene changes in β-catenin, cyclin D1, c-Myc, fibronectin, and MMP3 in β-catenin-knockdown LVFoxC1-RASFs (n=5), and corresponding control group (n=5) was set. Experiments were independently repeated three times. The data were expressed as mean ± SD. *p<0.05, **p<0.01, ***p<0.001, t-test.

The Wnt/β-catenin signaling pathway is important in RASFs. Although altering FoxC1 expression did not significantly change GSK3β and pGSK3β levels (**Figure 3A-B**), FoxC1 knockdown reduced the mRNA and total and nuclear protein levels of β-catenin in RASFs (**Figure 3A**). Furthermore, the mRNA and total protein levels of cyclin D1, c-Myc, fibronectin, and MMP3 in RASFs also decreased after FoxC1 knockdown (**Figure 3A**). Conversely, FoxC1 overexpression induced the opposite effects with regard to the expression patterns of β-catenin, cyclin D1, c-Myc, fibronectin, and MMP3 (**Figure 3B**).

**Figure 4:**
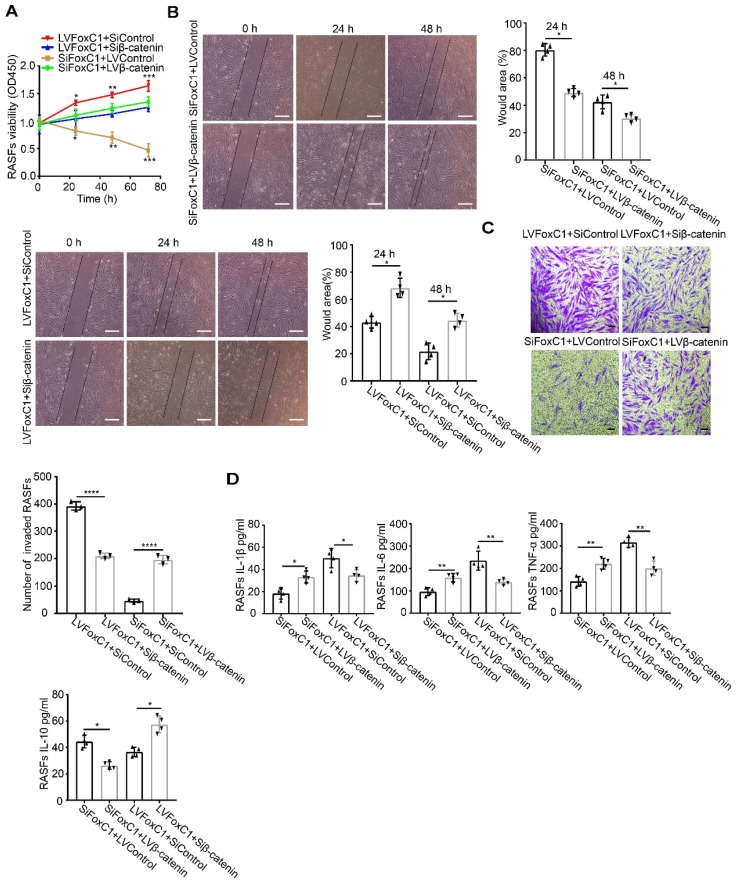
** FoxC1 induces pathological changes in RASFs through β-Catenin.** (A) The CCK-8 assay was used to evaluate cell proliferation after overexpression β-catenin SiFoxC1-RASFs (n=4), β-catenin- knockdown LVFoxC1-RASFs (n=4) in RASFs, and corresponding control groups (n=8) were set up. (B) Wound healing through RASFs (overexpression β-catenin SiFoxC1-RASFs (n=4), β-catenin-knockdown LVFoxC1-RASFs (n=4) and corresponding control groups (n=8) migration were observed sequentially (0 h, 24 h and 48 h) by microscopy. Original magnification ×100. The black lines were used to mark the general area of the wound. (C) Invasion of RASFs (overexpression β-catenin SiFoxC1-RASFs (n=3), β-catenin-knockdown LVFoxC1-RASFs (n=3) and corresponding control groups (n=6)) was determined by transwell assays (48 h). Original magnification ×200. (D) Inflammatory cytokines concentration in culture supernatant of RASFs (overexpression β-catenin SiFoxC1-RASFs (n=4), β-catenin-knockdown LVFoxC1-RASFs (n=4) and corresponding control groups (n=8)) was measured by ELISA. Experiments were independently repeated three times. The data were expressed as mean ± SD. *p<0.05, **p<0.01, ***p<0.001, ****p<0.0001, t-test. Scale bars: 100 μm.

To further clarify the role of β-catenin in FoxC1's regulation of RASF pathology, we transfected the β-catenin lentiviral overexpression vector (LVβ-catenin) in the case of FoxC1-inhibited RASFs and the β-catenin inhibitory siRNA (Siβ-catenin) in the case of FoxC1-overexpressed RASFs. We found that β-catenin overexpression increased the mRNA and total protein levels of cyclin D1, c-Myc, fibronectin, and MMP3 in SiFoxC1-transfected RASFs (**Figure 3C**). Conversely, β-catenin knockdown induced opposite changes with regard to the expression patterns of cyclin D1, c-Myc, fibronectin, and MMP3 in LVFoxC1-transfected RASFs (**Figure 3D**). Moreover, with regard to the viability, migration and invasion of RASFs, β-catenin overexpression attenuated the inhibitory effects of SiFoxC1, whereas β-catenin knockdown attenuated the enhancing effects of LVFoxC1 (**Figure 4A-C**). β-Catenin overexpression enhanced IL-1β, IL-6, and TNF-α production and decreased IL-10 production in SiFoxC1-transfected RASFs (**Figure 4D**), whereas β-catenin knockdown induced the opposite effects in LVFoxC1-transfected RASFs (**Figure 4D**).

### FoxC1 binds to the β-catenin promoter and activates β-catenin transcription in RASFs

**Figure 5:**
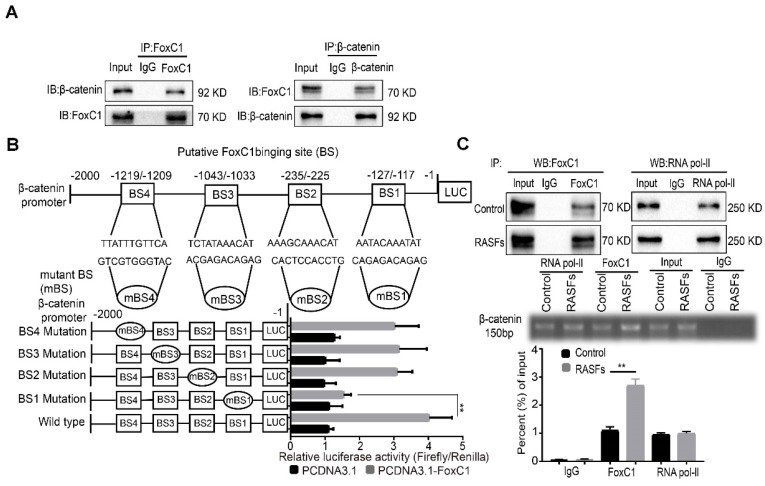
** FoxC1 binds to the β-catenin promoter and induces pathological changes in RASFs.** (A)The co-immunoprecipitation assay was used to detect the interaction of FoxC1 and β-catenin in RASFs (n=3). (B) Luciferase reporter assays were used to prove that the relationship between FoxC1 and β-catenin promoter in RASFs (n=3). (C) ChIP-qRT-PCR analysis confirmed the immunoprecipitation of FoxC1 and BS1 (n=3). Experiments were independently repeated three times. The data were expressed as mean ± SD. *p<0.05, **p<0.01, t-test, one-way ANOVA.

Because FoxC1 and β-catenin are closely related to each other in RASFs, we further investigated their relationship. First, the results of CoIP experiments indicate that there may be an interaction between FoxC1 and β-catenin proteins in RASFs (**Figure 5A**). To further explore whether FoxC1 directly regulates β-catenin transcription, luciferase reporter assays were performed, and the results showed that FoxC1 was able to bind to the promoter of β-catenin and promote its transcription (**Figure 5B**). Chromatin immunoprecipitation (ChIP) assays showed that an anti-FoxC1 antibody immunoprecipitated markedly more chromosomal DNA containing the β-catenin promoter than did the control IgG (**Figure 5C**).

Moreover, analysis of the β-catenin promoter sequence (from residues -2000 to +1 of the β-catenin sequence relative to the transcription start site) using JASPAR revealed four putative FoxC1 binding sites (BSs): BS1 (AATACAAATAT; residues -127 to -117), BS2 (AAAGCAAACAT; residues -235 to -225), BS3 (TCTATAAACAT; residues -1043 to -1033), and BS4 (TTATTTGTTCA; residues -1219 to -1209). Although mutations in BS2, BS3 and BS4 did not significantly affect luciferase activity, mutations in BS1 decreased FoxC1-induced luciferase activity (**Figure 5B**). The ChIP-qRT-PCR analysis confirmed the immunoprecipitation of FoxC1 and BS1, indicating that FoxC1 protein binds to the β-catenin promoter and exerts a significantly stronger effect in RASFs than in normal SFs (**Figure 5C**). These results suggested that FoxC1 directly activated β-catenin transcription in human SFs.

### MiR-141-3p binds to the FoxC1 3′UTR and influences the regulation of β-catenin by FoxC1

**Figure 6:**
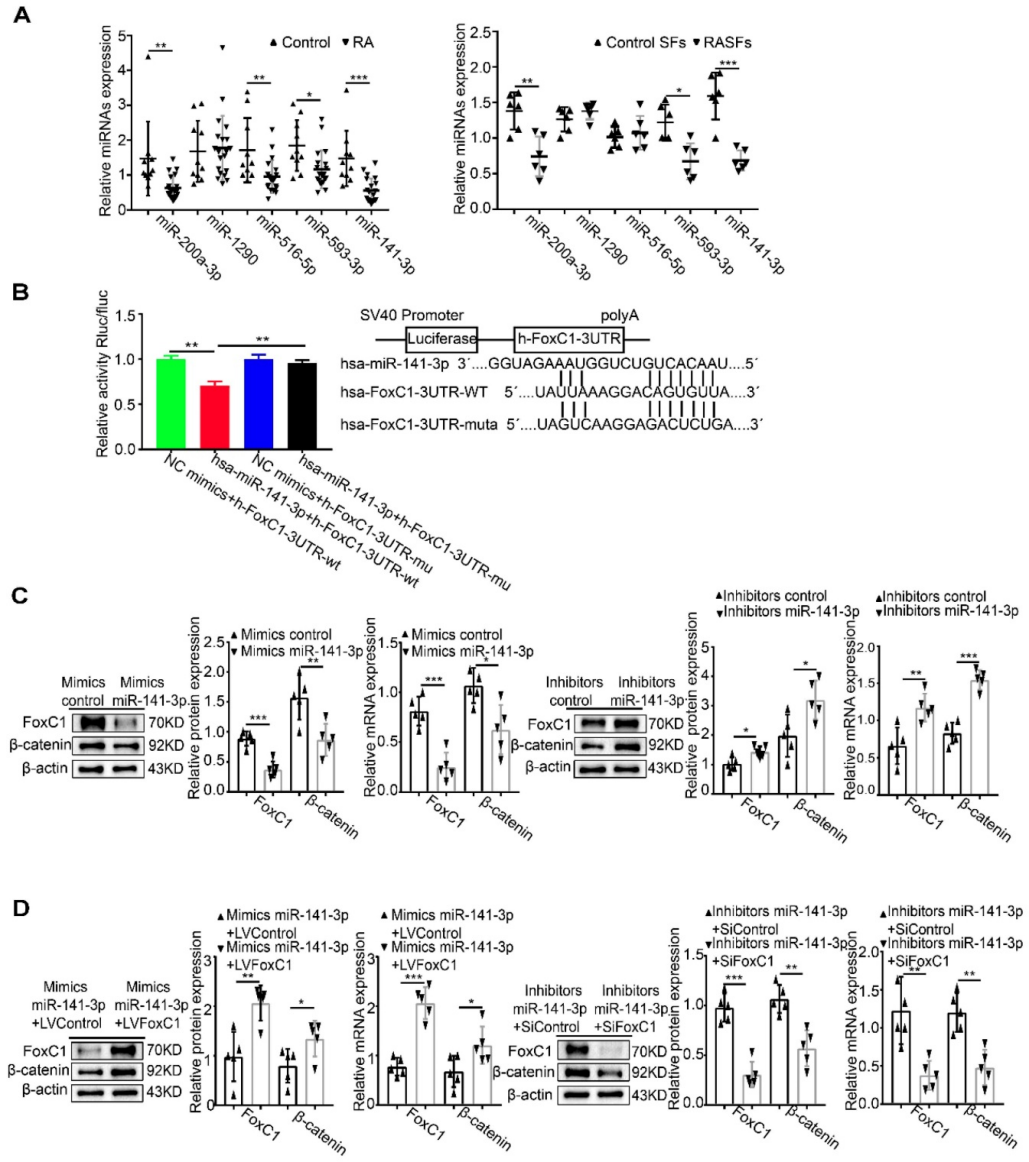
** MiR-141-3p binds to the FoxC1 3′UTR and influences the regulation of β-catenin by FoxC1.** (A) The expression of miRNAs in synovial tissues of RA patients (n=20) and RASFs (n=6) was detected by qRT-PCR, and corresponding control groups (control patients synovial tissue (n=10), control SFs (n=6)) were set up. (B) Dual-luciferase assay confirmed the binding of MiR-141-3p to FoxC1 3′UTR (n=3). (C) WB and qRT-PCR methods were used to detect protein and gene changes in FoxC1, β-catenin in overexpression miR-141-3p RASFs (n=5), miR-141-3p knockdown RASFs (n=5), and corresponding control groups (n=10) were set. (D) WB and qRT-PCR methods were used to detect protein and gene changes in FoxC1, β-catenin in overexpression miR-141-3p LVFoxC1-RASFs (n=5), miR-141-3p knockdown SiFoxC1-RASFs (n=5), and corresponding control groups (n=10) were set. Experiments were independently repeated three times. The data were expressed as mean ± SD. *p<0.05, **p<0.01, ***p<0.001, t-test, one-way ANOVA.

Combined with predictions from nine databases, it was found that miR-141-3p, miR-516b-5p, miR-200a-3p, miR-1290 and miR-593-3p may have binding sites with FoxC1. qRT-PCR analysis showed that the level of miR-141-3p was lower in both synovium (fold 2.66:1) and SFs (fold 2.31:1) from RA patients than in the control groups (**Figure 6A**). Next, the dual-luciferase reporter gene assay confirmed the binding of miR-141-3p to FoxC1 3′UTR in RASFs (**Figure 6B**). Importantly, we found that RASFs transfected with miR-141-3p mimic showed significantly downregulated mRNA and protein expression levels of FoxC1 and β-catenin, whereas transfection with miR-141-3p inhibitor resulted in significantly upregulated mRNA and protein expression levels of these molecules (**Figure 6C**).

To investigate whether miR-141-3p mediates β-catenin in RASFs by FoxC1, LVFoxC1 was used to upregulate the expression of FoxC1 expression in miR-141-3p mimic-treated RASFs, and SiFoxC1 was used to reduce the expression of FoxC1 in miR-141-3p inhibitor-treated RASFs. FoxC1 overexpression upregulated the expression of β-catenin mRNA and total protein in miR-141-3p mimic-treated RASFs, while FoxC1 knockdown caused the opposite changes in miR-141-3p inhibitor-treated RASFs (**Figure 6D**).

Based on the above research results, we surmised that the miR-141-3p/FoxC1/β-catenin axis plays a crucial role in the pathological mechanism of RA.

### Role of the miR-141-3p/FoxC1/β-catenin axis in LPS-induced inflammatory SFs

**Figure 7:**
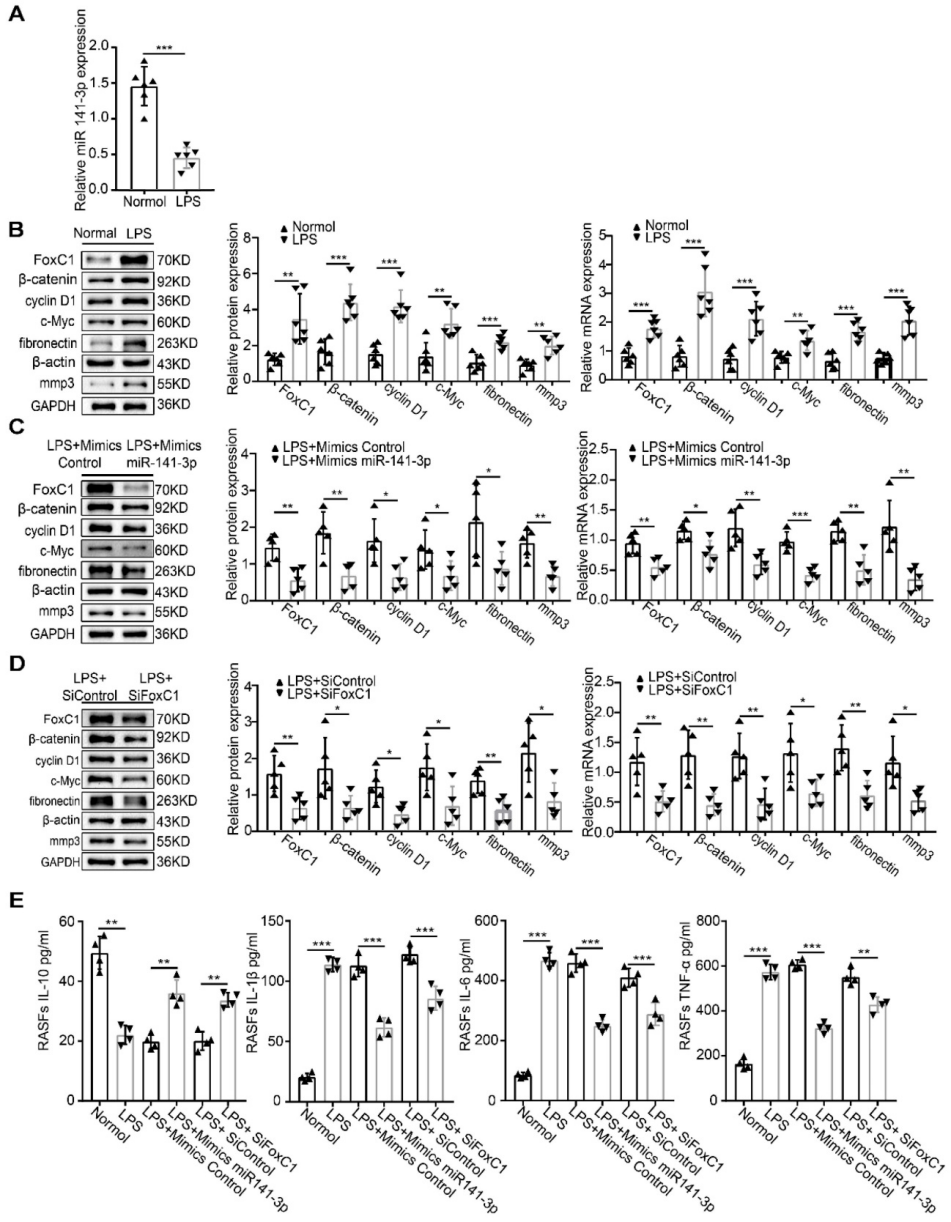
** Role of the miR-141-3p/FoxC1/β-catenin axis in LPS-induced inflammatory SFs.** (A) qRT-PCR was used to detect gene changes in miR-141-3p in LPS-induced SFs (n=6), and the corresponding control group (n=6) was set. (B) WB and qRT-PCR methods were used to detect protein and gene changes in FoxC1, β-catenin, cyclin D1, c-Myc, fibronectin, and MMP3 in LPS-induced SFs (n=6), and corresponding control group (n=6) was set. (C-D) WB and qRT-PCR methods were used to detect protein and gene changes in FoxC1, β-catenin, cyclin D1, c-Myc, fibronectin, and MMP3 in LPS-induced SFs (n=5), overexpression miR-141-3p LPS-induced SFs (n=5), FoxC1 knockdown LPS-induced SFs (n=5). (E) ELISA assay was used to evaluate the expression of inflammatory factors in SFs medium (n=4). Experiments were independently repeated three times. The data were expressed as mean ± SD. *p<0.05, **p<0.01, ***p<0.001, t-test.

**Figure 8:**
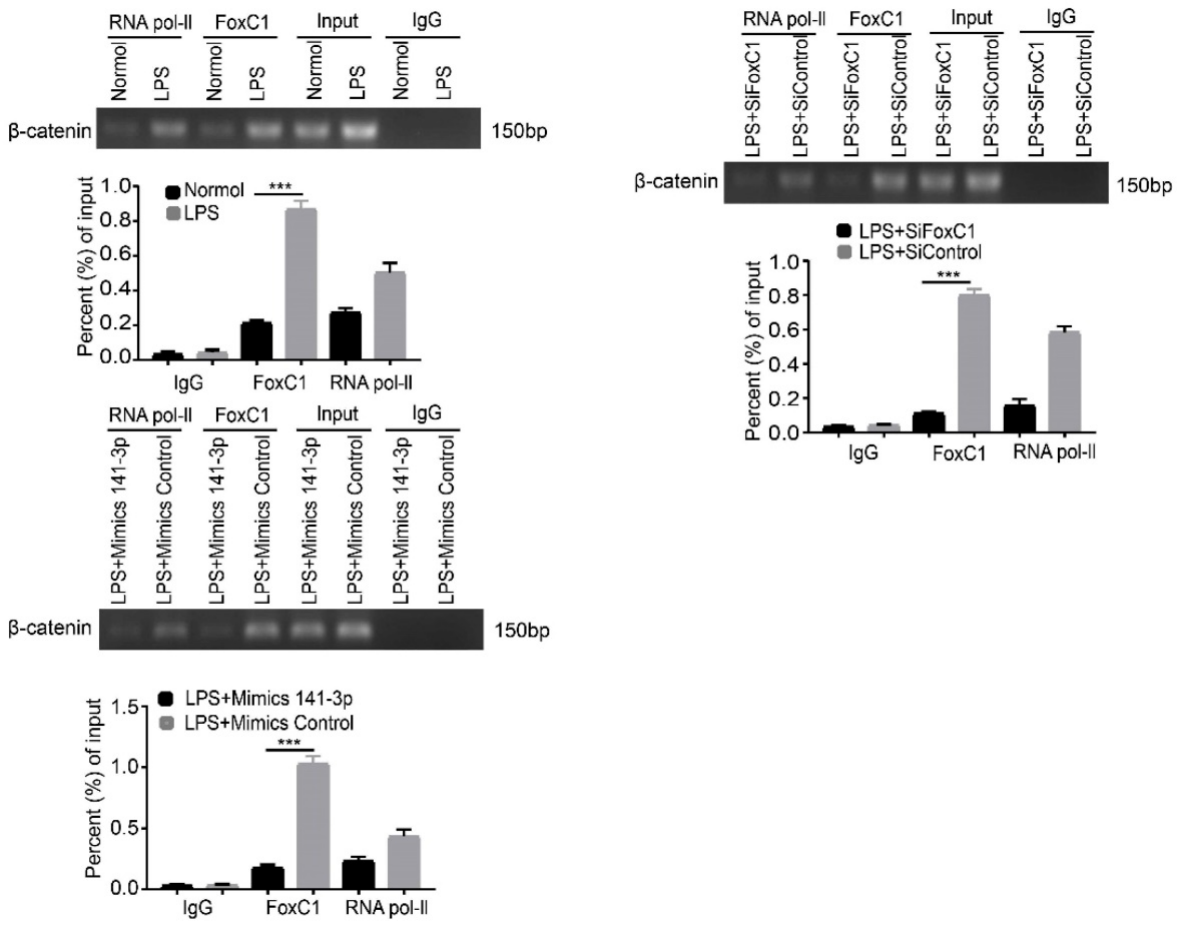
** FoxC1 binds to the β-catenin promoter and also had a significantly stronger effect in LPS-induced SFs.** ChIP and real-time PCR assays were used to measure FoxC1 bound to the β-catenin promoter in LPS-induced SFs (n=3), overexpression miR-141-3p LPS-induced SFs (n=3) and FoxC1 knockdown LPS-induced SFs (n=3). Experiments were independently repeated three times. The data were expressed as mean ± SD. *p<0.05, **p<0.01, ***p<0.001, t-test

We induced normal SFs with LPS and similarly found that miR-141-3p was significantly decreased at the gene level (**Figure 7A**). In contrast, levels of FoxC1, β-catenin, cyclin D1, c-Myc, fibronectin, and MMP3 mRNA and total protein were increased (**Figure 7B**). However, these adverse effects were alleviated by miR-141-3p overexpression, FoxC1 knockdown or β-catenin knockdown (**Figure 7C-D**). Importantly, the levels of the inflammatory cytokines IL-1β, IL-6, and TNF-α were significantly increased, while that of IL-10 was decreased, after LPS induction (**Figure 7E**). Data detected by ChIP and qRT-PCR assays indicated the presence of FoxC1 binding to the β-catenin promoter and exerted a significantly stronger effect in LPS-induced SFs than in normal SFs; furthermore, this binding capacity was inhibited by miR-141-3p overexpression, FoxC1 knockdown or β-catenin knockdown (**Figure 8**). These data suggest that miR-141-3p/FoxC1/β-catenin may still be present in the LPS-induced inflammatory SFs model.

### Intra-ankle injection of a miR-141-3p agomir/FoxC1-specific siRNA hinders CIA development in rats

**Figure 9:**
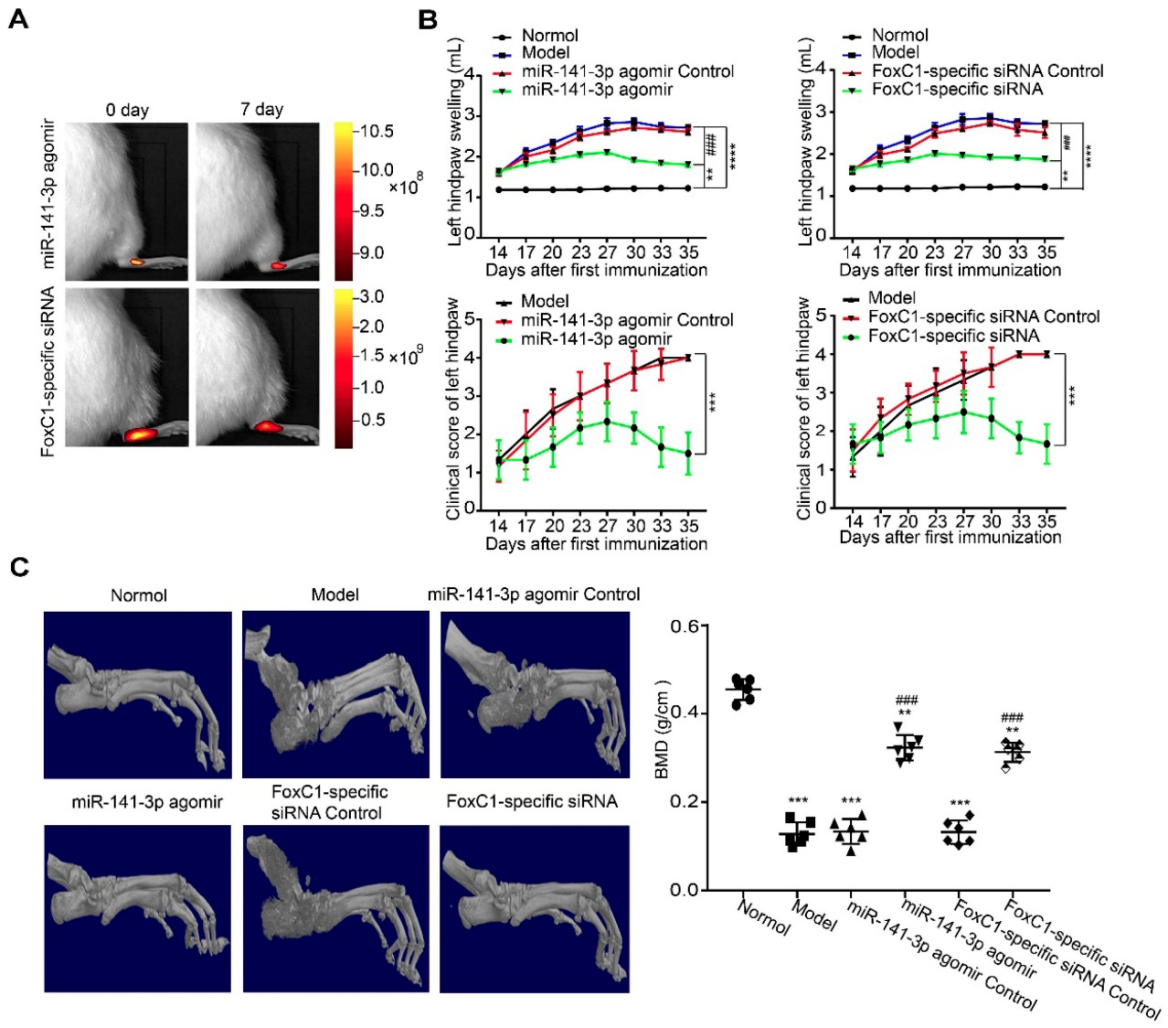
** Intra-ankle injection of a miR-141-3p agomir /FoxC1-specific siRNA hinders CIA development in rats.** (A) In vivo imaging results showed that miR-141-3p agomir and FoxC1-specific siRNA could be expressed in the ankle joint of rats for at least 7 days. (B) Clinical arthritis score and ankle joint swelling in the arthritic rats were evaluated (n=6). (C) Micro-CT was used to measure bone destruction and bone density (n=6). The data were expressed as mean ± SD. *p<0.05, **p<0.01, ***p<0.001,****p<0.0001 versus the normal group, ### p<0.001 versus the model group. one-way ANOVA.

**Figure 10:**
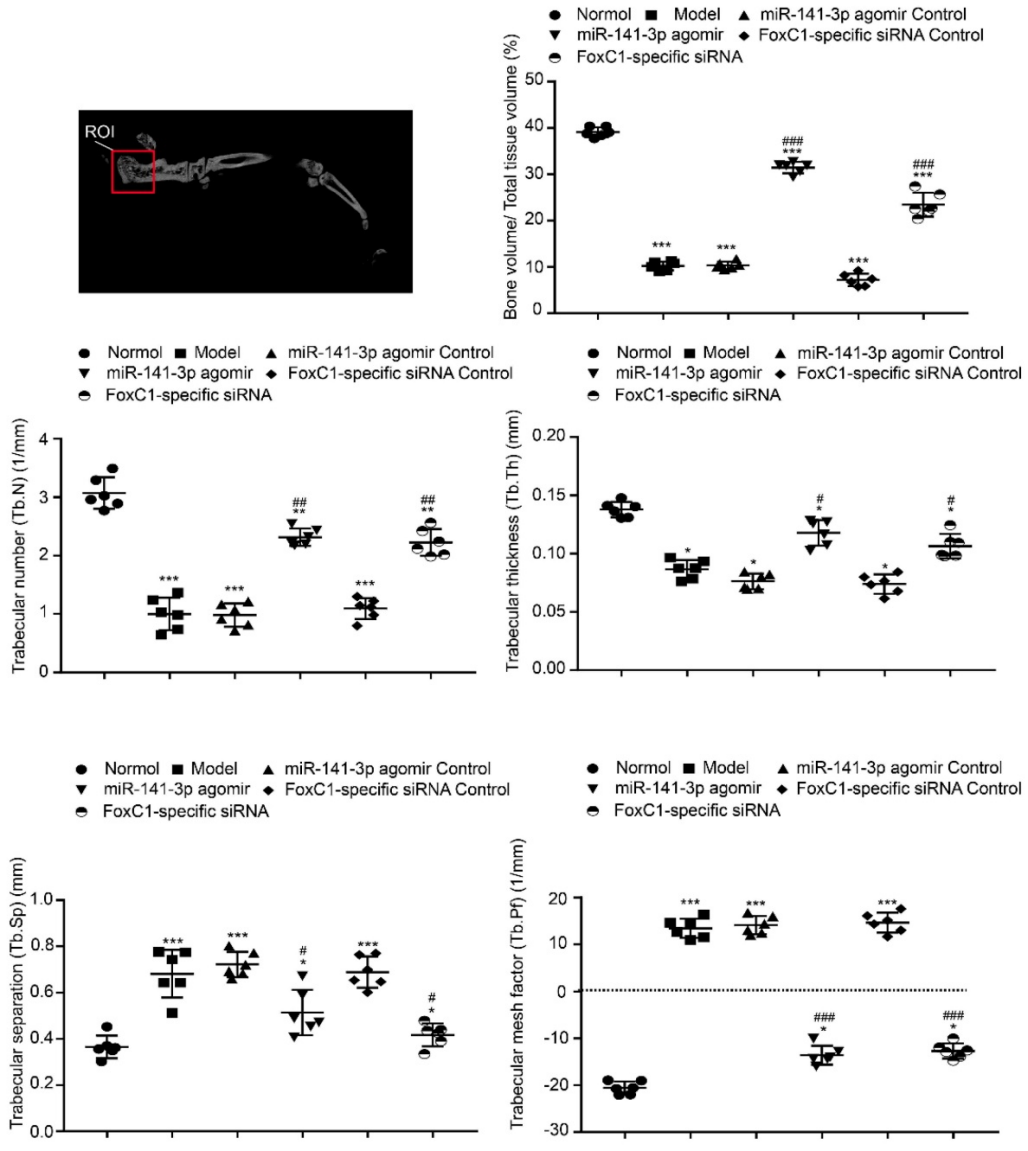
** Morphology and iconography study of rat treatment by miR-141-3p agomir/FoxC1-specific siRNA.** The bone trabecular of the left calcaneus was chosen as region of interest (ROI). Micro-CT was used to measure ankle bone parameters. The data were expressed as mean ± SD. *p<0.05, **p<0.01, ***p<0.001 versus the normal group, #p<0.05, ##p<0.01, ###p<0.001 versus the model group. One-way ANOVA.

To clarify whether FoxC1 or miR-141-3p could be a therapeutic target for RA in vivo, we evaluated the potential clinical efficacy of FoxC1 knockdown and miR-141-3p overexpression using a rat model of RA (CIA). The rats received intra-articular injections of sicontrol, negative control miRNA, siFoxC1, or miR-141-3p agomir into their left hind ankle joints. To determine the duration of FoxC1 or miR-141-3p agomir in the ankle, we used in vivo imaging to confirm that siFoxC1 and miR-141-3p were still expressed 7 days after the first injection (**Figure 9A**). Intra-articular injection of siFoxC1 or miR-141-3p agomir significantly improved both the clinical arthritis score and ankle joint swelling in arthritic rats compared with those in control rats (**Figure 9B**). Micro-CT data showed that bone erosion was significantly reduced in rats injected with siFoxC1 or miR-141-3p agomir compared with the control groups (**Figure 9C**). The calcaneus was selected as the ROI, and related parameters including BMD percentage (**Figure 9C**), BV/TV, Tb.N, Tb.Th, Tb.Sp, and Tb.Pf were analyzed. The analysis results showed that both siFoxC1 and miR-141-3p agomir groups showed improvements in all parameters compared with the control group (**Figure 10**).

**Figure 11:**
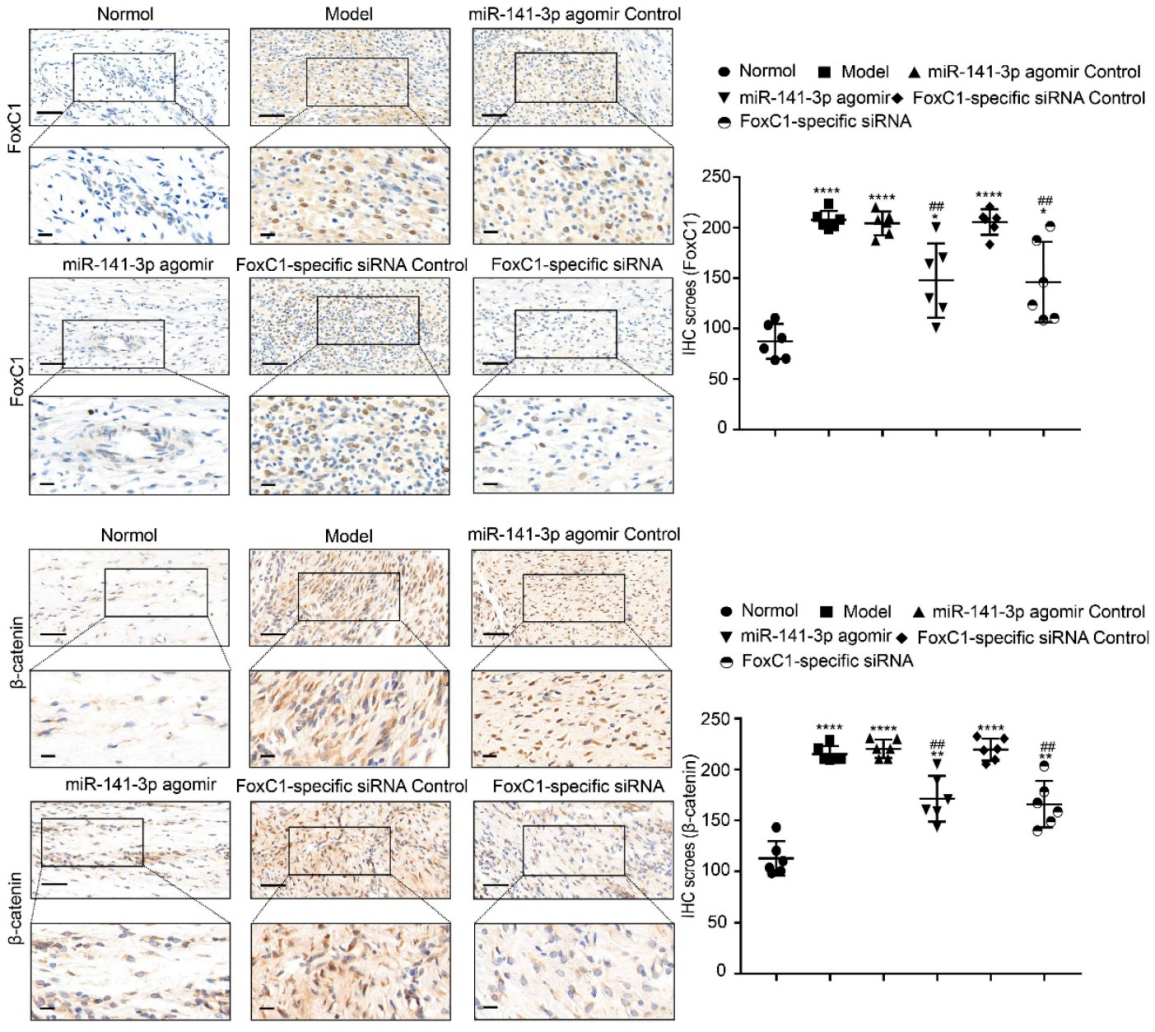
** Expression levels of FoxC1 and β-catenin after injection of miR-141-3p agomir/FoxC1-specific siRNA in CIA rats.** Immunohistochemistry and IHC scores were used to evaluate the expression of FoxC1 and β-catenin after injection of miR-141-3p agomir/FoxC1-specific siRNA. Original magnification ×200, original magnification ×400. Experiments were independently repeated three times. The data were expressed as mean ± SD. *p<0.05, **p<0.01, **** p<0.0001 versus the normal group, ##p<0.01 versus the model group. One-way ANOVA. Scale bars: 50μm.

**Figure 12:**
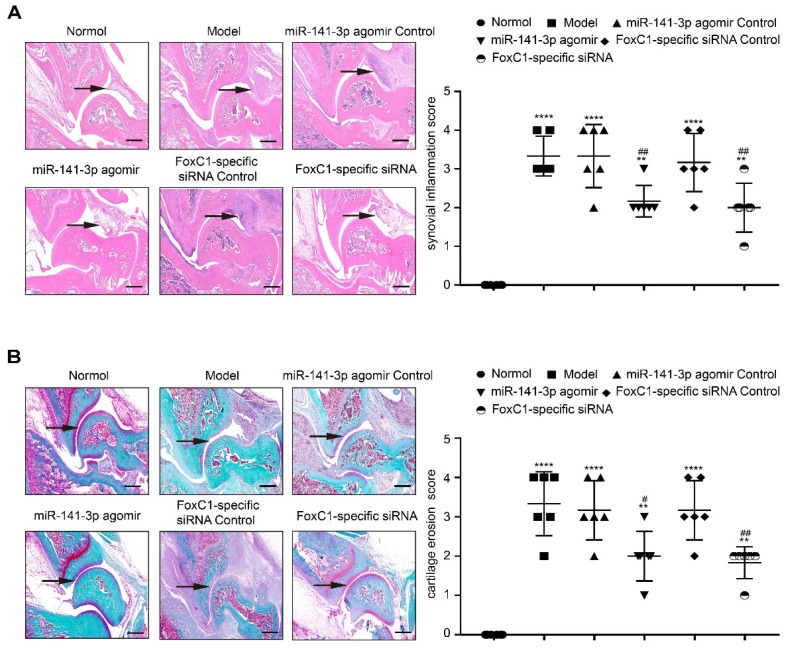
** Intra-ankle injection of a miR-141-3p agomir /FoxC1-specific siRNA hinders CIA development in rats.** (A) HE staining and synovial inflammation score were used to assess synovial proliferation and inflammation (n=6). The black arrow was used to mark the synovial position of the ankle. (B) The degree of cartilage damage in the ankle was evaluated by Safranin O/Fast Green staining and cartilage erosion score (n=6). The arrows were used to mark the cartilage position. Black arrows were used to mark the cartilage surface of the ankle. All values were expressed as mean ± SD. **p<0.01, ****p<0.0001 versus the normal group, #p<0.05, ##p<0.01 versus the model group. one-way ANOVA. Original magnification ×20. Scale bars: 500 μm.

**Figure 13:**
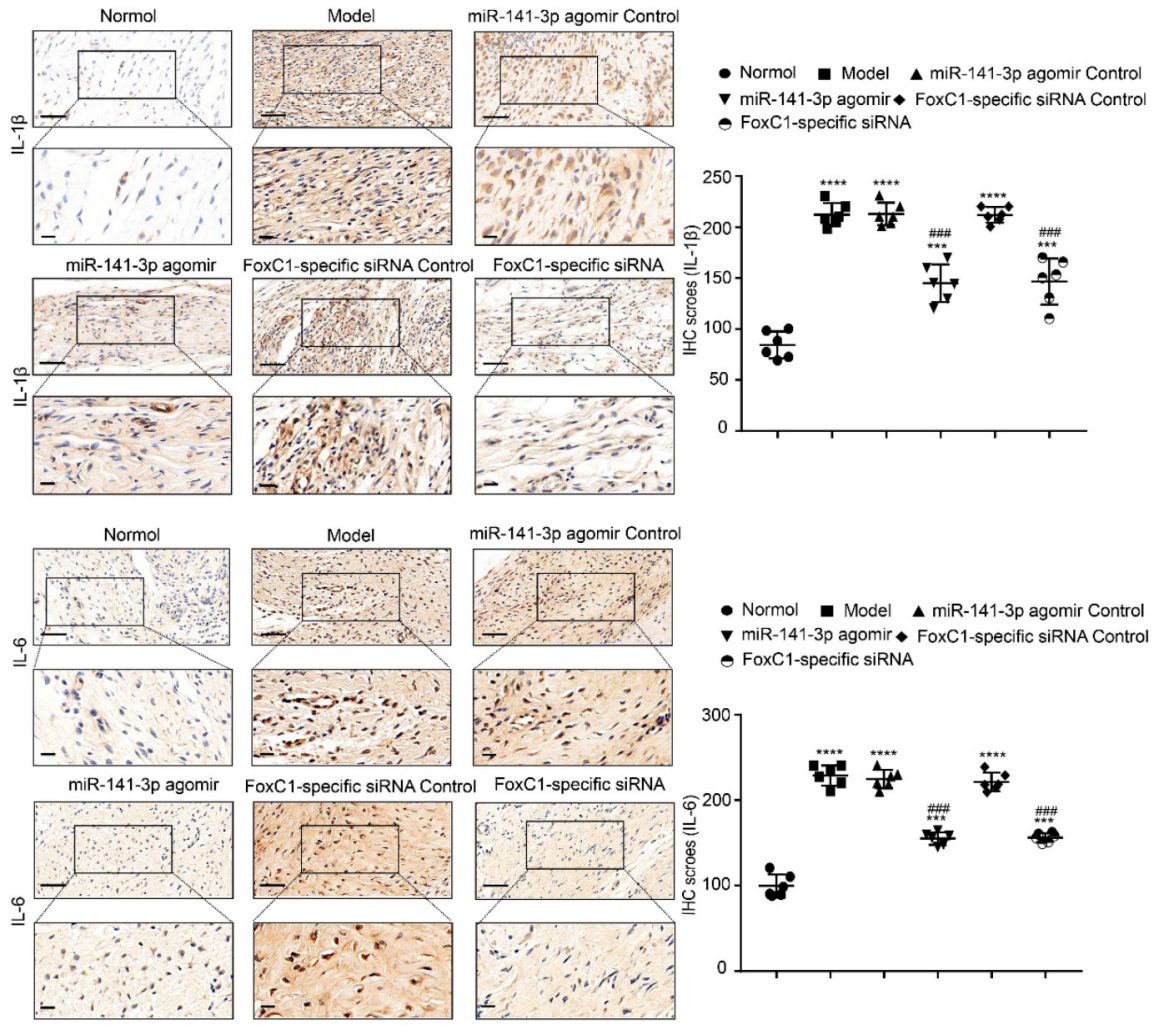
** IL-1β and IL-6 expression in CIA rats after treatment by miR-141-3p agomir/FoxC1-specific siRNA.** Immunohistochemistry and IHC scores were used to evaluate the expression of IL-1β and IL-6 after injection of miR-141-3p agomir/FoxC1-specific siRNA. Original magnification ×200, original magnification ×400. Experiments were independently repeated three times. The data were expressed as mean ± SD. ***p<0.001, **** p<0.0001 versus the normal group, ###p<0.001, ####p<0.0001 versus the model group. One-way ANOVA. Scale bars: 50μm.

**Figure 14:**
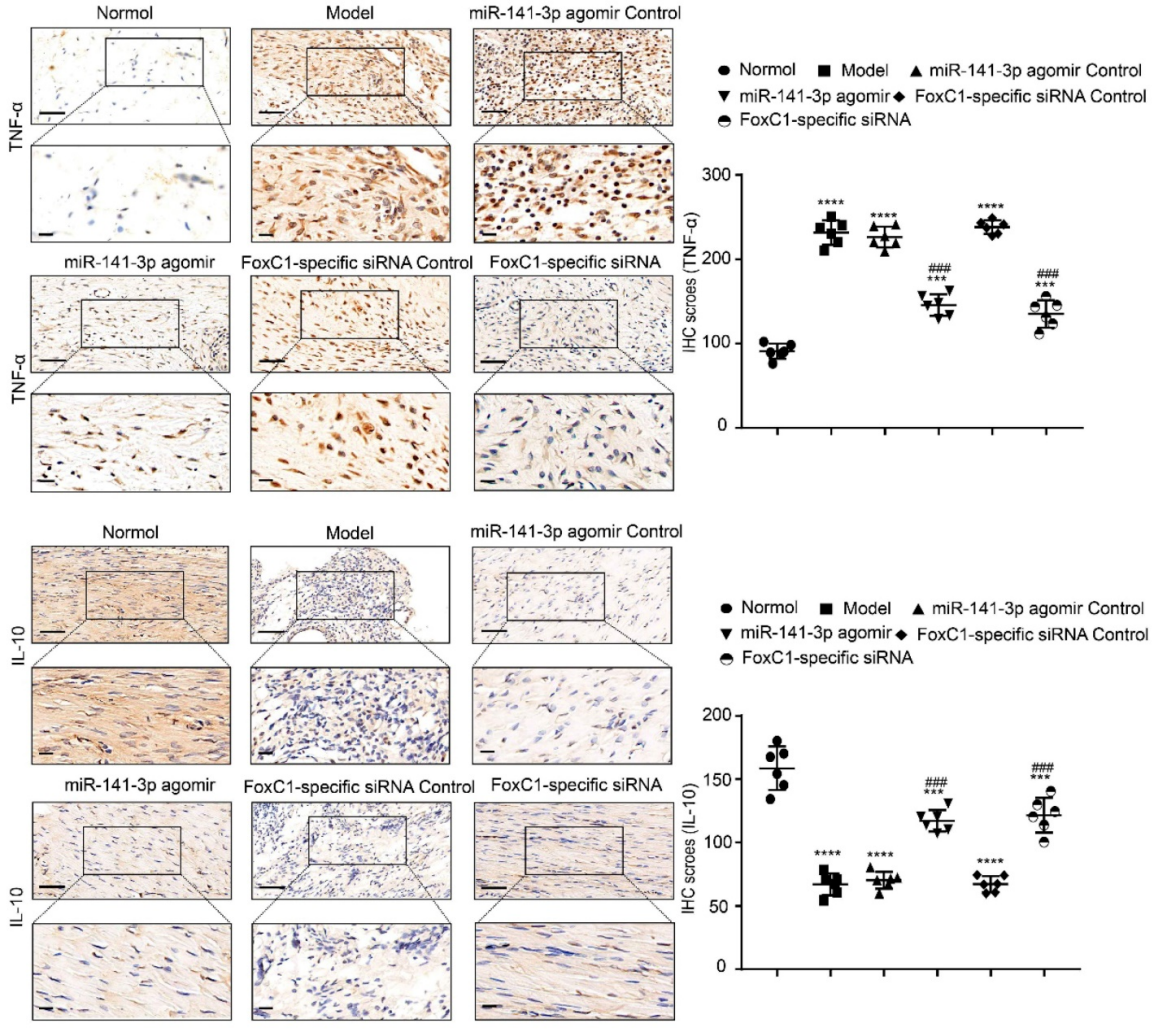
** TNF-α and IL-10 expression in CIA rats after treatment by FoxC1-specific siRNA/miR-141-3p agomir.** Immunohistochemistry and IHC scores were used to evaluate the expression of TNF-α and IL-10 after the injection of miR-141-3p agomir/FoxC1-specific siRNA. Original magnification ×200, original magnification ×400. Experiments were independently repeated three times. The data were expressed as mean ± SD. ***p<0.001, **** p<0.0001 versus the normal group, ###p<0.001, ####p<0.0001 versus the model group. One-way ANOVA. Scale bars: 50μm.

IHC results showed that the expression of FoxC1 and β-catenin in the synovial tissue of the ankle joint cavity was significantly decreased in the CIA rats injected with siFoxC1 or miR-141-3p agomir compared with the control group (**Figure 11**). Surprisingly, the siFoxC1 and miR-141-3p agomir groups exhibited thinner synovial membranes and less cell infiltration in contrast to the synovitis aggravation in the control group (**Figure 12A**). Moreover, siFoxC1 and miR-141-3p agomir significantly decreased cartilage destruction compared with that observed with the control compounds (**Figure 12B**). IHC also confirmed that the levels of the proinflammatory cytokines IL-1β, IL-6, and TNF-α were lower and those of the anti-inflammatory cytokine IL-10 were higher in the siFoxC1- or miR-141-3p agomir-injected rats compared with the corresponding levels in the control compound rats (**Figure 13, 14**).

## Discussion

Although FoxC1 is known as an essential factor in cancer pathology, its regulatory contribution to tumor-like characteristics and its role in the pathological progression of RA have not been thoroughly discussed. Now, we have studied these topics in detail in vivo and in vitro and revealed a new molecular mechanism. Our results demonstrate, for the first time, a novel role of the miR-141-3p/FoxC1/β-catenin axis in regulating RA functional plasticity in vivo and in vitro.

RASFs display an aggressive phenotype with a tumor-like appearance and play key roles in tissue destruction during the pathogenesis of RA by producing inflammatory cytokines 54-56 and ECM-degrading proteases^57^, ^58^. Our data show for the first time that FoxC1 is highly expressed in the synovium and SFs of RA patients and CIA rats and may metastasize to the nucleus. Previous studies have indicated that FoxC1 is overexpressed in human cancer and acts as an oncogene to promote proliferation and metastasis. Additionally, it is well known that inflammatory cytokines, such as IL-1β, IL-6 and TNF-α, are upregulated and that the anti-inflammatory cytokine IL-10 is downregulated during the pathogenesis of RA. Moreover, in our study, inhibition of FoxC1 expression resulted in decreased IL-1β, IL-6, and TNF-α production and increased IL-10 production, while FoxC1 overexpression induced the opposite effects. A recent study revealed that FoxC1 promotes cell proliferation by upregulating PI3K/AKT signaling in RA in an inflammation-dependent manner; unfortunately, the inflammatory cytokine-associated mechanism has not been explored 59. Therefore, our data support an underappreciated role of FoxC1 in promoting proliferation, migration, and proinflammatory cytokine (IL-1β, IL-6, TNF-α) production and inhibiting anti-inflammatory cytokine secretion (IL-10) in RASFs.

Next, we explored the molecular mechanism underlying the regulatory effects of FoxC1 on RASF properties. The Wnt/β-catenin signaling pathway has been proven to be associated with the occurrence and development of various diseases in a number of previous studies 60-62, and it also plays an important role in the pathological mechanism of RA 63-65. When this pathway is activated, large amounts of β-catenin enter the nucleus, and nuclear β-catenin forms complexes with TCF/LEF to regulate target gene expression 66, 67. Interestingly, our current results revealed that FoxC1 knockdown reduced β-catenin levels and downregulated cyclin D1, c‑Myc, fibronectin, and MMP3 expression in RASFs and suggested that these regulatory effects are unrelated to GSK3β. Moreover, we found that β-catenin overexpression reversed the inhibitory effects of FoxC1 knockdown on cyclin D1, c‑Myc, fibronectin, and MMP3 expression and on RASF proliferation, migration, invasion and inflammatory response (by decreasing IL-1β, IL-6, and TNF-α and increasing IL-10 levels). Cyclin D1 and c-Myc are common downstream molecules of the β-catenin/TCF4 complex and are related to cell proliferation, migration and invasion in a variety of tumor mechanisms 68-70. In addition, as components of the ECM, fibronectin and MMP3 are highly expressed during cell migration and invasion, chronic inflammation and tissue destruction, as observed in autoimmune disorders such as RA 65, 71, 72. A previous study on podocyte injury found that shRNA-mediated knockdown of β-catenin abolished the upregulation of fibronectin and matrix metalloproteinase-9 (MMP9) induced by advanced oxidation protein products 73. Imai et al. found that β-catenin nuclear translocation can immediately cause the upregulated expression of catabolic genes (MMP3, ADAMTS-4) in chondrocytes 74.

These findings suggest that β-catenin is closely related to the production of fibronectin and MMP3. There is also evidence of correlations between inflammatory cytokine expression and β-catenin. Lin JC et al. found that overexpression of β-catenin led to an increase in the inflammatory cytokines TNF-α and IL-8 and activated cardiomyocytes through the NF-κB signaling pathway 75. β-catenin molecular targeted therapy is an underlying inflammatory and fibrotic cardiomyocyte 75. Furthermore, Bo Gao et al. found that MALAT1 significantly downregulated the expression of IL-6, IL-1β and TNF-α and upregulated the expression of IL-10 in RASFs by promoting β-catenin promoter methylation and inhibiting β-catenin expression 76. All of these findings indicate that β-catenin might play an important role in the proliferation, migration, invasion, and secretion of inflammatory factors in RASFs.

The luciferase reporter assays in this study revealed that FoxC1 promoted β-catenin expression in RASFs by enhancing β-catenin promoter activity. The CoIP assays confirmed the existence of binding between the FoxC1 and β-catenin proteins in RASFs, and the ChIP and qRT-PCR assays confirmed the binding of FoxC1 to the β-catenin promoter (from sites -127 to -117) in RASFs. Thus, our data further suggest that FoxC1 causes a series of pathological changes in RASFs by regulating β-catenin via FoxC1 binding to the β-catenin promoter to promote the transcription of β-catenin. In this manner, FoxC1 may stabilize β-catenin and has a positive effect on Wnt/β-catenin signaling.

MiR-141-3p is a member of the miR-200 family, which is considered to include important negative regulators of cancer cell proliferation, migration and invasion, and has been found to regulate immune cells during the inflammatory response 77, 78. Our data showed for the first time that miR-141-3p expression levels were significantly reduced in both synovium and SFs in RA patients. More importantly, our results showed that miR-141-3p overexpression reduced the expression of FoxC1, β-catenin, cyclin D1, c-Myc, fibronectin, and MMP3, decreased the secretion of the proinflammatory cytokines IL-1β, IL-6, and TNF-α and increased the production of IL-10 in RASFs, while miR-141-3p knockdown reversed these effects. The dual-luciferase reporter assay results showed that FoxC1 was a target gene of miR-141-3p. These results show, for the first time, that miR-141-3p may affect the proliferation, migration, invasion, and secretion of inflammatory cytokines in RASFs by regulating FoxC1. LPS, a natural immune reaction regulator, has been used extensively to study the mechanisms and regulation of immune reactions 79, 80. Our results also indicated that LPS can induce an artificial RA cell model and that the miR-141-3p/FoxC1/β-catenin axis regulates the underlying pathological changes.

Silencing FoxC1 or enhancing miR-141-3p through intra-articular injection of siFoxC1/miR-141-3p agomir suppressed experimentally induced arthritis in rats by inhibiting SF proliferation and inflammation. The fact that siFoxC1 or miR-141-3p agomir could reduce inflammation of synovial tissue and prevent further destruction of articular cartilage and bone suggests that FoxC1 and miR-141-3p are potential therapeutic targets for RA. Although the contribution of FoxC1 and miR-141-3p to the immune system as a whole is unclear, inhibiting FoxC1 or inducing miR-141-3p could be a potential treatment for RA. However, we must recognize that systemic inhibition of FoxC1 or miR-141-3p may result in more severe dysfunction, and complete blockade of the signaling proteins with specific chemical inhibitors is impossible.

Of course, our research has certain limitations. Although synovial tissues from RA patients diagnosed based on the criteria of the American College of Rheumatology were included, we cannot completely exclude the possibility that these tissues were from osteoarthritis patients to some extent. The mechanism underlying how all of the effectors downstream of β-catenin (cyclin D1, c‑Myc, fibronectin, MMP3, IL-1β, IL-6, TNF-α and IL-10) are regulated in RA still needs to be explored in the future. In addition, it should be recognized that the joint cavity contains a variety of cells and tissues and that siFoxC1 or miR-141-3p agomir may also alleviate the progression of arthritis in CIA rats by acting on other cells in the joint such as chondrocytes and osteocytes.

In this study, our results suggest that the miR-141-3p/FoxC1/β-catenin signaling axis may contribute to the pathogenesis of RA by regulating the proliferation of, migration of and inflammatory cytokine production from SFs. Thus, we propose that FoxC1 and miR-141-3p might be potential novel targets for nonimmunosuppressive-based RA therapies. Further studies are needed to clarify whether FoxC1 and mir-141-3p can be used as diagnostic markers and prognostic indicators for RA, and how to apply FoxC1 and miR-141-3p to clinical treatment.

## Supplementary Material

Supplementary figure, tables, materials.

Supplementary movie

## Abbreviations

RA: rheumatoid arthritis
SFs: synovial fibroblasts
FoxC1: Forkhead box protein C1
microRNA (miR)-141-3p: (miR)-141-3p
MMP3: matrix metalloproteinase 3
IL-1β: interleukin-1β
mRNA: messenger RNA
TNF-α: tumor necrosis factor-α
H&E: hematoxylin and eosin
CCK-8: Counting Kit-8 assays
ROI: the region of interest
ChIP: chromatin immunoprecipitation
CoIP: coimmunoprecipitation
CIA: collagen-induced arthritis
LVFoxC1: overexpression of FoxC1
siFoxC1: inhibition of FoxC1
LVβ-catenin: overexpression of β-catenin
siβ-catenin: inhibition of β-catenin
miR-141-3p mimic: overexpression of miR-141-3p
miR-141-3p inhibitor: inhibition of miR-141-3p
