# Supplementary material for: Identification of a novel microRNA-141-3p/Forkhead box C1/β-catenin axis associated with rheumatoid arthritis synovial fibroblast function in vivo and in vitro
Source: Theranostics. 2020 Apr 6;10(12):5412–34. doi: 10.7150/thno.45214 (PMC7196314; doi:10.7150/thno.45214)
Supplement: Supplementary file 1 — Supplementary figure, tables, materials. [file thnov10p5412s1.pdf]

Supplementary Figure and Figure Legends

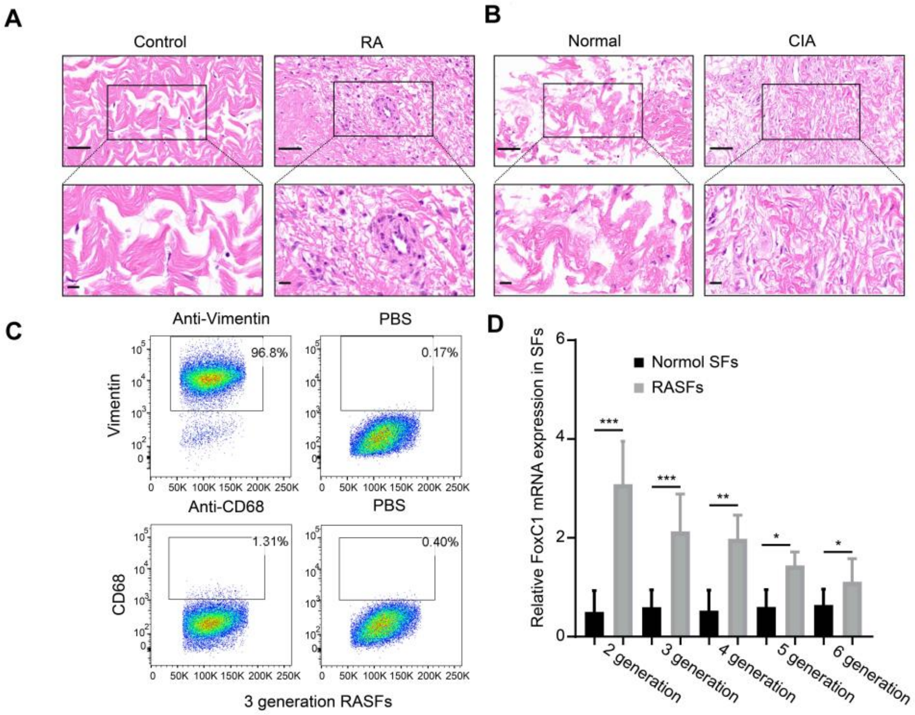

Supplementary Figure 1. FoxC1 and β-catenin are significantly upregulated in the synovium and SFs of RA patients and CIA rats. (A-B) The synovial tissue morphology of RA patients (n = 20), CIA rats (n = 6), and the corresponding control groups were identified by HE staining. Original magnification ×200, original magnification ×400. (C) CD68 and vimentin were detected by flow cytometry in 3 generations RASFs. CD68 was used to label synovial macrophages. Vimentin was used to label synovial fibroblasts. (D) FoxC1 expression in different generations of SFs (normal SFs (n=3) and RASFs (n=3)) was detected by qRT-PCR. Experiments were independently repeated three times. The data were expressed as mean ± SD. *p<0.05, **p<0.01, ***p<0.001, t-test. Scale bars: 50 μm.

Supplementary Table

Supplementary Table 1. General patient information (n=30)

Patients	Rheumatoid arthritis(n=20)	Amputation due to trauma (n=4)	Requiring arthroscopic surgery(n=6).
Age in years, mean (SD)	50.9 (13.7)	31.7(5.9)	21.4(10.6)
Female, n (%)	15(75)	2(50)	4(66.7)
Duration of rheumatoid arthritis (year), mean (SD)	9.5(8.5)		
RF positive, n (%)	16(80)		
Anti-CCP positive n (%)	13(60)		
ESR (mm/hr), mean (SD)	36.9(25.2)		
CRP (mg/l), mean (SD)	20.1(20.4)		
DAS28-ESR, mean (SD)	5.6(1.5)		
HAQ, mean (SD)	1.5(0.7)		
NSAID, n(%)	18(90)		
DMARD, n(%)	12(60)		
Prednisolone, n(%)	8(40)		
Previous biological therapy n(%)	1(5)		

RF, rheumatoid factor; DMARD, disease-modifying anti-rheumatic drugs; Anti-CCP, anti-cyclic citrullinated peptide; ESR, erythrocyte sedimentation rate; CRP, c-reactive protein; DAS28-ESR, disease activity score for 28-joint counts based on the esr; HAQ, health assessment questionnaire; NSAID, non-steroidal anti-inflammatory drug; mm, millimeter; hr,hour; mg, milligram; l, liter

Supplementary Table 2. Experimental correlation primers.

Gene	Sequences
Human FoxC1 sense	5'-CAGCATCCGCCACAACCTCT-3'
Human FoxC1 antisense	5'-GCAGCCTGTCCTTCTCCTCCT-3'
Rat FoxC1 sense	5'-ACGGCACAACCTCTCGCTTAATG-3'
Rat FoxC1 antisense	5'-CTTGTCCTTCACCGCGTCCTTC-3'
Human β-Catenin sense	5'-GGCTCTTGTGCGTACTGTCCTTC-3'
Human β-Catenin antisense	5'-GCTTCTTGGTGTCGGCTGGTC-3'
Rat β-Catenin sense	5'-GTTGCTCCACTCCAGGAATGAAGG-3'

Rat β-Catenin antisense	5'- GCACCAATGTCCAGTCCGAGATC-3'
Human GSK-3β sense	5'-AGGAGAACCCAATGTTTCGTAT-3'
Human GSK-3β antisense	5'- ATCCCCTGGAAATATTGGTTGT-3'
Human c-Myc sense	5'-CGAGGAGAATGTCAAGAGGCGAAC-3'
Human c-Myc antisense	5'- GCTTGGACGGACAGGATGTATGC-3'
Human cyclin D1 sense	5'- TACCGCCTCACACGCTTCCTC-3'
Human cyclin D1 antisense	5'-ACCTCCTCCTCCTCCTCTTCCTC-3'
Human fibronectin sense	5'- ATGCAACGATCAGGACACAAGGAC-3'
Human fibronectin antisense	5'-TGCCTCTCACACTTCCACTCTCC-3'
Human MMP3 sense	5'-CGAGGAGAATGTCAAGAGGCGAA -3'
Human MMP3 antisense	5'-GCTTGGACGGACAGGATGTATGC-3'
β-actin sense	5’- AGAAGATCTGGCACCACACC-3’
β-actin antisense	5’- GAACCAGGAGTTAAGAACACG-3’
miR-141-3p sense	5’- CGGCTAACACTGTCTGGTAAAGATGG -3’
miR -593-3p sense	5’- CGTGTCTCTGCTGGGGTTTCT -3’
miR -516b-5p sense	5’-GGCATCTGGAGGTAAGAAGCACTTT-3’
miR 1290 sense	5’- CGCGTGGATTTTTGGATCAGGGA -3’
miR -200a-3p sense	5’-GCGTAACACTGTCTGGTAACGATGT -3’
U6 sense	5’-CTCGCTTCGGCAGCACATATACT-3’

Supplementary Table 3. siRNAs, miR-141-3p mimic and inhibitor sequences used in this work

Gene	Sequences
Human siFoxC1 sense	5'-CCAGUGAACGGGAAUAGUAUU-3'
Human siFoxC1 antisense	5'-UACUAUUCCCGUUCACUGGUU-3'
Rat siFoxC1 sense	5'-GCCACAAGAUUACAAGAAAUU-3'
Rat siFoxC1 antisense	5'-UUUCUUGUAAUCUUGUGGCUU-3'
Human siβ-Catenin sense	5'-UCACCUCGUGGUACCUGAAUU-3'
Human siβ-Catenin antisense	5'-UUCAGGUACCACGAGGUGAUU-3'
miR-141-3p mimic sense	5'-UAACACUGUCUGGUAAAGAUGG-3'
miR-141-3p mimic antisense	5'-CCAUCUUUACCAGACAGUGUUA-3'
miR-141-3p inhibtor	5'-CCAUCUUUACCAGACAGUGUUA-3'

Supplementary materials for luciferase reporter assay

Cloning details including oligonucleotide sequence for Luciferase reporter assay

Double luciferase assay confirmed the existence of binding sites

between miR-141-3p and FoxC1

Vector and target gene information

1. The psi-check2 vector map is as follows:

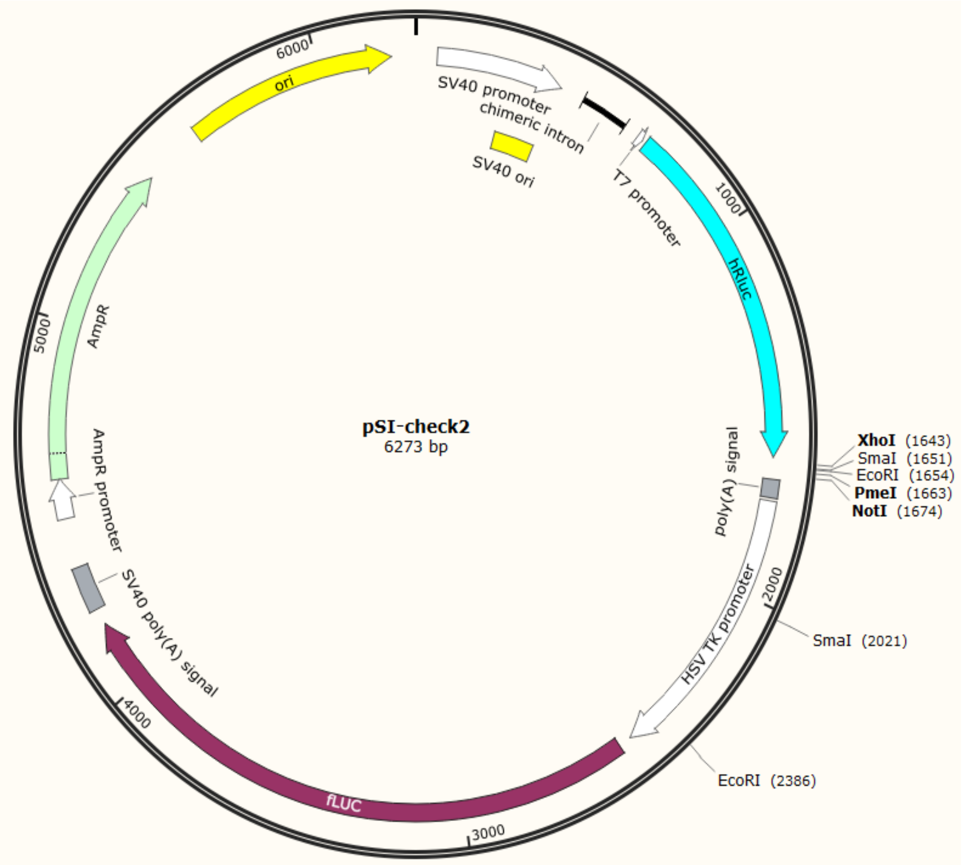

2. Human-FoxC1and hsa-miR-141-3p sequence information

h-FOXC1-3UTR-wt:
ctcgagCACACCCTCAAAGCCGAACTAAATCGAACCCCAAAGCAGGAAAAGCTA
AAGGAACCCATCAAGGCAAAATCGAAACTAAAAAAAAAAAAATCCAATTAAAAAA
AACCCCTGAGAATATTCACCACACCAGCGAACAGAATATCCCTCCAAAAATTCA
GCTCACCAGCACCAGCACGAAGAAAACTCTATTTTCTTAACCGATTAATTCAGA
GCCACCTCCACTTTGCCTTGTCTAAATAAACAAACCCGTAAACTGTTTTATACA
GAGACAGCAAAATCTTGGTTTATTAAAGGACAGTGTTACTCCAGATAACACGTA
AGTTTCTTCTTGCTTTTCAGAGACCTGCTTTCCCCTCCTCCCGTCTCCCCTCTC
TTGCCTTCTTCCTTGCCTCTCACCTGTAAGATATTATTTTATCCTATGTTGAAG
GGAGGGGGAAAGTCCCCGTTTATGAAAGTCGCTTTCTTTTTATTCATGGACTTG
TTTTAAAATGTAAATTGCAAgcggccgc

h-FOXC1-3UTR-mu:
ctcgagCACACCCTCAAAGCCGAACTAAATCGAACCCCAAAGCAGGAAAAGCTA
AAGGAACCCATCAAGGCAAAATCGAAACTAAAAAAAAAAAAATCCAATTAAAAAA

AACCCCTGAGAATATTCACCACACCAGCGAACAGAATATCCCTCCAAAAATTCA
GCTCACCAGCACCAGCACGAAGAAAACTCTATTTTCTTAACCGATTAATTCAGA
GCCACCTCCACTTTGCCTTGTCTAAATAAACAAACCCGTAAACTGTTTTATACA
GAGACAGCAAAATCTTGGTTTAgTcAAGGAgAcTcTgACTCCAGATAACACGTA
AGTTTCTTCTTGCTTTTCAGAGACCTGCTTTCCCCTCCTCCCGTCTCCCCTCTC
TTGCCTTCTTCCTTGCCTCTCACCTGTAAGATATTATTTTATCCTATGTTGAAG
GGAGGGGGAAAGTCCCCGTTTATGAAAGTCGCTTTCTTTTTATTCATGGACTTG
TTTTAAAATGTAAATTGCAAgcggccgc

>hsa-miR-141-3p MIMAT0000432
UAACACUGUCUGGUAAAGAUGG

Double luciferase assay confirmed the existence of a binding site between FoxC1 and β-catenin promoter

Vector and target gene information

1. The psi-check2 vector map is as follows:

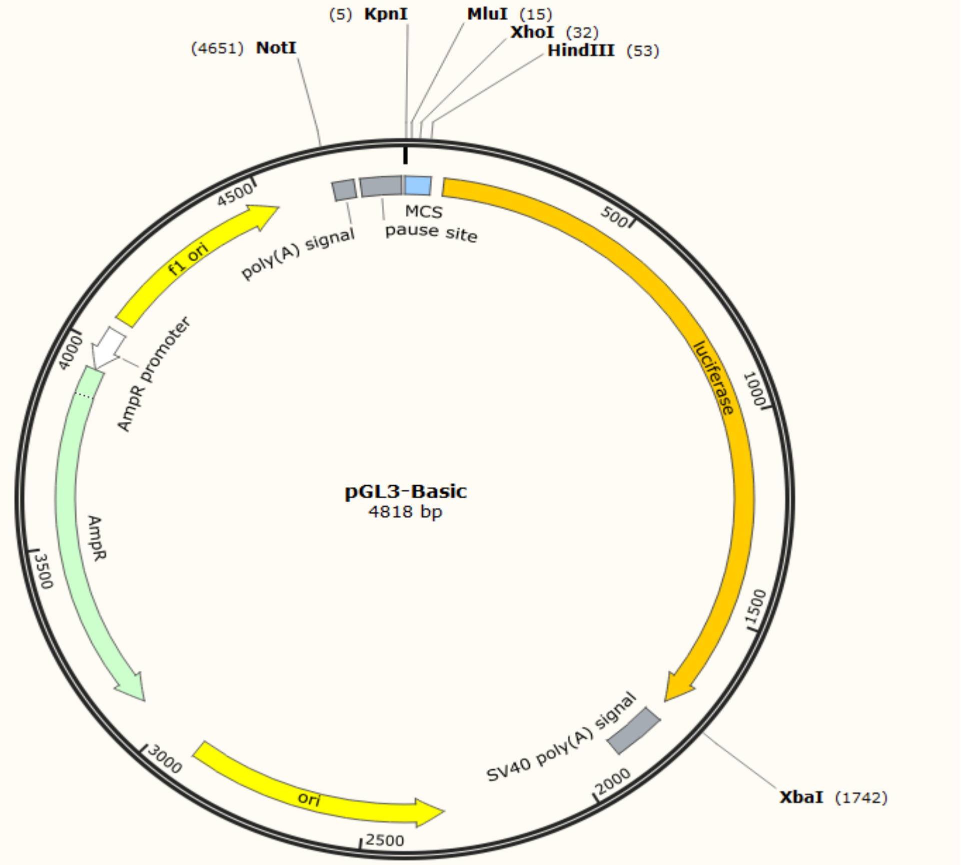

2. Human- β-catenin-promoter sequence information

h-β-catenin-pro-wt：

TCAGTAGGGATTAAAAATCATAATTCTAAATTTAAAGTATTTAAATTATAGAAAAATGATTCCAT
TATTTTGGTAATGGAACAGAGAAATACAAATATAGAAACGTTTTTATCCTTTAGATCTTCTAATG
GTGCAGATATTTCTTAACCAATTTCAAGAGTGCCTTATTCAAAAACAAAAAACCTGCACATAGAA
AAAGCAAACATGTTTTTTTAAAAAAAGCCCTGTAAAAGTGGTTAATCTGTCGTCTGATTTTCAAT
GTATGACTTTAATCAATTGCATATTCCTTAATTCTGCAAATGACAATTAAAATTAGGAATCAGTA
CCTGAAAACGCCATTTATACTTTTGAGTTTATATTAGAAACACGGTTTTGATGAAATACCTTTTT
CGGTTCAATCTTTTTGAATTGTGACCACAACCAATAGCTACACTTAGATTCTACAGAATTAGTAG
GAAAGATACAAATGAGAAGGTCTTTCTAGACAATGGATCTCACTGAACATCATCTTAGTGTGTAG
ACTTTTTCCACTGCAGTGGCCCACATGTGCTATGGTGACAGACGGCAGTTGGCATTACCACTTAT
ATAGTAAAATATCTTTAAGACAAAAATAAAAGTTATTTAAAGAATACGCTGGCCCTGAAACATGA
GCTGTGCCTTAAACTACATATCCTACCTCCATGAGTGAATGAATAAGTGGGTTTTCCGCCTGCAT
CTATGTGTTAAGACCTATTGTTTGCTTGTATTAATTGTAGTCCTGGGATAAAAGCACAGAGGTAA
CTTTCACTGCTGCTTTTTGTACTCTCTCCAATGTTTTGGAGGAAAAATAAGCACAAACAATAGCC
TAGAGAAACTGAATCGATCATACTTGTTGCAGCTTCGACAAACGTCAATTTTGCTGCATTAGAAT
GGGAAACATTTTTCAGTCTATTGAAATGAATTACAAACGTTTTTAAATAGAAAATTAGTTAAAAA
ATTGGAGGCTGCTTAATCGATAGCTTTCTCTATAAACATACTTGGATTTCACAAATAAGTAATAC
CGTAAAAATCTTCTTCTCCAAAGAAAAATCCCCACAAATAAATCTATTGATACCTAGTGACAAGT
GGAACCAGATAAAAATGGAATCTATAAGAATTAACCTAATTGACAGCGCTCTGGAGCTAATCCAT
TTCCATTAGTTATTTGTTCACAGTAGGTACTCCTAAGGACTTGTTGAATTGCGGGCTTGGCGCCC
GTTCTACGGAGAGTTCACAGCCTTCGTGAGTGGGGACAGAAGGCGGCTCGGCCCGGTGATTCAGG
TCGAAATTCAAGCTGAACAGCCTGCTGAGAGGTGGGATCCACCATCCGGACAGTGGGGGGCTTTG
GGGGTGCTGTGAGACTGGGCTGCGACCCAGGTCCAGCAGGGAGTGTGCGGCACAGACCACAAGGT
CGGCGAGGCCCCCTAACCCGCGCCCGGCCGGGAACCCGCAGACCAGCGACGGGGCAGCTGCGGGG
CCAGGAGCGCCCCAAGACGGGCGGGCGCTGAACCCGAGCCCCTGCCGCCGCCCTGGCCCCGAACT
TCCGCCCTCCCAGGACCTGTCCCGGCCGCCCCGAGCGGTACTCGAAGGCCGGGGCCGAGATGCCA
CCTTCCGCAGGCCGCGGGAAAGGCGCGCCGAGTCCTGCAGCTGCTCTCCCGGTTCGGGAAACGCG
CGGGGCGGGGGCGTCGGGCTTGGGACAGGGGAGGATACCAGGGCCACCTTCCCCAACCCAGGCCG
CGGGGGCCCGGCCTCCCCGATGCAGACCACAGCGCCCTCACGGGCTGCCCTCAGGCCGCGCAGCG
GGCAGCCGCCAGCCGTCACCCCGGGGAGCGTCCGTGGGGTGCCCAGGCACCCCACCCCGGCCCGG
GGCGCTCAGACGGCAGCAGACTGCTGGGCGGCGCGGGGACTACTTTCCACCGCCCCCTCGCGCCC
CGCCCCTTGTCCTCGCGCGGCGGAACGCTCCGCGCTGCGCCGGTGGCGGC

h-β-catenin-pro-mu1：

TCAGTAGGGATTAAAAATCATAATTCTAAATTTAAAGTATTTAAATTATAGAAAAATGATTCCAT
TATTTTGGTAATGGAACAGAGAcAgAgAcAgAgAGAAACGTTTTTATCCTTTAGATCTTCTAATG
GTGCAGATATTTCTTAACCAATTTCAAGAGTGCCTTATTCAAAAACAAAAAACCTGCACATAGAA
AAAGCAAACATGTTTTTTTAAAAAAAGCCCTGTAAAAGTGGTTAATCTGTCGTCTGATTTTCAAT
GTATGACTTTAATCAATTGCATATTCCTTAATTCTGCAAATGACAATTAAAATTAGGAATCAGTA
CCTGAAAACGCCATTTATACTTTTGAGTTTATATTAGAAACACGGTTTTGATGAAATACCTTTTT
CGGTTCAATCTTTTTGAATTGTGACCACAACCAATAGCTACACTTAGATTCTACAGAATTAGTAG
GAAAGATACAAATGAGAAGGTCTTTCTAGACAATGGATCTCACTGAACATCATCTTAGTGTGTAG
ACTTTTTCCACTGCAGTGGCCCACATGTGCTATGGTGACAGACGGCAGTTGGCATTACCACTTAT
ATAGTAAAATATCTTTAAGACAAAAATAAAAGTTATTTAAAGAATACGCTGGCCCTGAAACATGA
GCTGTGCCTTAAACTACATATCCTACCTCCATGAGTGAATGAATAAGTGGGTTTTCCGCCTGCAT

CTATGTGTTAAGACCTATTGTTTGCTTGTATTAATTGTAGTCCTGGGATAAAAGCACAGAGGTAA
CTTTCACTGCTGCTTTTTGTACTCTCTCCAATGTTTTGGAGGAAAAATAAGCACAAACAATAGCC
TAGAGAAACTGAATCGATCATACTTGTTGCAGCTTCGACAAACGTCAATTTTGCTGCATTAGAAT
GGGAAACATTTTTCAGTCTATTGAAATGAATTACAAACGTTTTTAAATAGAAAATTAGTTAAAAA
ATTGGAGGCTGCTTAATCGATAGCTTTCTCTATAAACATACTTGGATTTCACAAATAAGTAATAC
CGTAAAAATCTTCTTCTCCAAAGAAAAATCCCCACAAATAAATCTATTGATACCTAGTGACAAGT
GGAACCAGATAAAAATGGAATCTATAAGAATTAACCTAATTGACAGCGCTCTGGAGCTAATCCAT
TTCCATTAGTTATTTGTTCACAGTAGGTACTCCTAAGGACTTGTTGAATTGCGGGCTTGGCGCCC
GTTCTACGGAGAGTTCACAGCCTTCGTGAGTGGGGACAGAAGGCGGCTCGGCCCGGTGATTCAGG
TCGAAATTCAAGCTGAACAGCCTGCTGAGAGGTGGGATCCACCATCCGGACAGTGGGGGGCTTTG
GGGGTGCTGTGAGACTGGGCTGCGACCCAGGTCCAGCAGGGAGTGTGCGGCACAGACCACAAGGT
CGGCGAGGCCCCCTAACCCGCGCCCGGCCGGGAACCCGCAGACCAGCGACGGGGCAGCTGCGGGG
CCAGGAGCGCCCCAAGACGGGCGGGCGCTGAACCCGAGCCCCTGCCGCCGCCCTGGCCCCGAACT
TCCGCCCTCCCAGGACCTGTCCCGGCCGCCCCGAGCGGTACTCGAAGGCCGGGGCCGAGATGCCA
CCTTCCGCAGGCCGCGGGAAAGGCGCGCCGAGTCCTGCAGCTGCTCTCCCGGTTCGGGAAACGCG
CGGGGCGGGGGCGTCGGGCTTGGGACAGGGGAGGATACCAGGGCCACCTTCCCCAACCCAGGCCG
CGGGGGCCCGGCCTCCCCGATGCAGACCACAGCGCCCTCACGGGCTGCCCTCAGGCCGCGCAGCG
GGCAGCCGCCAGCCGTCACCCCGGGGAGCGTCCGTGGGGTGCCCAGGCACCCCACCCCGGCCCGG
GGCGCTCAGACGGCAGCAGACTGCTGGGCGGCGCGGGGACTACTTTCCACCGCCCCCTCGCGCCC
CGCCCCTTGTCCTCGCGCGGCGGAACGCTCCGCGCTGCGCCGGTGGCGGC

h-β-catenin-pro-mu2：
TCAGTAGGGATTAAAAATCATAATTCTAAATTTAAAGTATTTAAATTATAGAAAAATGATTCCAT
TATTTTGGTAATGGAACAGAGAAATACAAATATAGAAACGTTTTTATCCTTTAGATCTTCTAATG
GTGCAGATATTTCTTAACCAATTTCAAGAGTGCCTTATTCAAAAACAAAAAACCTGCACATAGAA
cAcTcCaCcTgGTTTTTTTAAAAAAAGCCCTGTAAAAGTGGTTAATCTGTCGTCTGATTTTCAAT
GTATGACTTTAATCAATTGCATATTCCTTAATTCTGCAAATGACAATTAAAATTAGGAATCAGTA
CCTGAAAACGCCATTTATACTTTTGAGTTTATATTAGAAACACGGTTTTGATGAAATACCTTTTT
CGGTTCAATCTTTTTGAATTGTGACCACAACCAATAGCTACACTTAGATTCTACAGAATTAGTAG
GAAAGATACAAATGAGAAGGTCTTTCTAGACAATGGATCTCACTGAACATCATCTTAGTGTGTAG
ACTTTTTCCACTGCAGTGGCCCACATGTGCTATGGTGACAGACGGCAGTTGGCATTACCACTTAT
ATAGTAAAATATCTTTAAGACAAAAATAAAAGTTATTTAAAGAATACGCTGGCCCTGAAACATGA
GCTGTGCCTTAAACTACATATCCTACCTCCATGAGTGAATGAATAAGTGGGTTTTCCGCCTGCAT
CTATGTGTTAAGACCTATTGTTTGCTTGTATTAATTGTAGTCCTGGGATAAAAGCACAGAGGTAA
CTTTCACTGCTGCTTTTTGTACTCTCTCCAATGTTTTGGAGGAAAAATAAGCACAAACAATAGCC
TAGAGAAACTGAATCGATCATACTTGTTGCAGCTTCGACAAACGTCAATTTTGCTGCATTAGAAT
GGGAAACATTTTTCAGTCTATTGAAATGAATTACAAACGTTTTTAAATAGAAAATTAGTTAAAAA
ATTGGAGGCTGCTTAATCGATAGCTTTCaCgAgAcAgAgACTTGGATTTCACAAATAAGTAATAC
CGTAAAAATCTTCTTCTCCAAAGAAAAATCCCCACAAATAAATCTATTGATACCTAGTGACAAGT
GGAACCAGATAAAAATGGAATCTATAAGAATTAACCTAATTGACAGCGCTCTGGAGCTAATCCAT
TTCCATTAGTTATTTGTTCACAGTAGGTACTCCTAAGGACTTGTTGAATTGCGGGCTTGGCGCCC
GTTCTACGGAGAGTTCACAGCCTTCGTGAGTGGGGACAGAAGGCGGCTCGGCCCGGTGATTCAGG
TCGAAATTCAAGCTGAACAGCCTGCTGAGAGGTGGGATCCACCATCCGGACAGTGGGGGGCTTTG
GGGGTGCTGTGAGACTGGGCTGCGACCCAGGTCCAGCAGGGAGTGTGCGGCACAGACCACAAGGT

CGGCGAGGCCCCCTAACCCGCGCCCGGCCGGGAACCCGCAGACCAGCGACGGGGCAGCTGCGGGG
CCAGGAGCGCCCCAAGACGGGCGGGCGCTGAACCCGAGCCCCTGCCGCCGCCCTGGCCCCGAACT
TCCGCCCTCCCAGGACCTGTCCCGGCCGCCCCGAGCGGTACTCGAAGGCCGGGGCCGAGATGCCA
CCTTCCGCAGGCCGCGGGAAAGGCGCGCCGAGTCCTGCAGCTGCTCTCCCGGTTCGGGAAACGCG
CGGGGCGGGGGCGTCGGGCTTGGGACAGGGGAGGATACCAGGGCCACCTTCCCCAACCCAGGCCG
CGGGGGCCCGGCCTCCCCGATGCAGACCACAGCGCCCTCACGGGCTGCCCTCAGGCCGCGCAGCG
GGCAGCCGCCAGCCGTCACCCCGGGGAGCGTCCGTGGGGTGCCCAGGCACCCCACCCCGGCCCGG
GGCGCTCAGACGGCAGCAGACTGCTGGGCGGCGCGGGGACTACTTTCCACCGCCCCCTCGCGCCC
CGCCCCTTGTCCTCGCGCGGCGGAACGCTCCGCGCTGCGCCGGTGGCGGC

h-β-catenin-pro-mu3：

TCAGTAGGGATTAAAAATCATAATTCTAAATTTAAAGTATTTAAATTATAGAAAAATGATTCCAT
TATTTTGGTAATGGAACAGAGAAATACAAATATAGAAACGTTTTTATCCTTTAGATCTTCTAATG
GTGCAGATATTTCTTAACCAATTTCAAGAGTGCCTTATTCAAAAACAAAAAACCTGCACATAGAA
AAAGCAAACATGTTTTTTTAAAAAAAGCCCTGTAAAAGTGGTTAATCTGTCGTCTGATTTTCAAT
GTATGACTTTAATCAATTGCATATTCCTTAATTCTGCAAATGACAATTAAAATTAGGAATCAGTA
CCTGAAAACGCCATTTATACTTTTGAGTTTATATTAGAAACACGGTTTTGATGAAATACCTTTTT
CGGTTCAATCTTTTTGAATTGTGACCACAACCAATAGCTACACTTAGATTCTACAGAATTAGTAG
GAAAGATACAAATGAGAAGGTCTTTCTAGACAATGGATCTCACTGAACATCATCTTAGTGTGTAG
ACTTTTTCCACTGCAGTGGCCCACATGTGCTATGGTGACAGACGGCAGTTGGCATTACCACTTAT
ATAGTAAAATATCTTTAAGACAAAAATAAAAGTTATTTAAAGAATACGCTGGCCCTGAAACATGA
GCTGTGCCTTAAACTACATATCCTACCTCCATGAGTGAATGAATAAGTGGGTTTTCCGCCTGCAT
CTATGTGTTAAGACCTATTGTTTGCTTGTATTAATTGTAGTCCTGGGATAAAAGCACAGAGGTAA
CTTTCACTGCTGCTTTTTGTACTCTCTCCAATGTTTTGGAGGAAAAATAAGCACAAACAATAGCC
TAGAGAAACTGAATCGATCATACTTGTTGCAGCTTCGACAAACGTCAATTTTGCTGCATTAGAAT
GGGAAACATTTTTCAGTCTATTGAAATGAATTACAAACGTTTTTAAATAGAAAATTAGTTAAAAA
ATTGGAGGCTGCTTAATCGATAGCTTTCaCgAgAcAgAgACTTGGATTTCACAAATAAGTAATAC
CGTAAAAATCTTCTTCTCCAAAGAAAAATCCCCACAAATAAATCTATTGATACCTAGTGACAAGT
GGAACCAGATAAAAATGGAATCTATAAGAATTAACCTAATTGACAGCGCTCTGGAGCTAATCCAT
TTCCATTAGTTATTTGTTCACAGTAGGTACTCCTAAGGACTTGTTGAATTGCGGGCTTGGCGCCC
GTTCTACGGAGAGTTCACAGCCTTCGTGAGTGGGGACAGAAGGCGGCTCGGCCCGGTGATTCAGG
TCGAAATTCAAGCTGAACAGCCTGCTGAGAGGTGGGATCCACCATCCGGACAGTGGGGGGCTTTG
GGGGTGCTGTGAGACTGGGCTGCGACCCAGGTCCAGCAGGGAGTGTGCGGCACAGACCACAAGGT
CGGCGAGGCCCCCTAACCCGCGCCCGGCCGGGAACCCGCAGACCAGCGACGGGGCAGCTGCGGGG
CCAGGAGCGCCCCAAGACGGGCGGGCGCTGAACCCGAGCCCCTGCCGCCGCCCTGGCCCCGAACT
TCCGCCCTCCCAGGACCTGTCCCGGCCGCCCCGAGCGGTACTCGAAGGCCGGGGCCGAGATGCCA
CCTTCCGCAGGCCGCGGGAAAGGCGCGCCGAGTCCTGCAGCTGCTCTCCCGGTTCGGGAAACGCG
CGGGGCGGGGGCGTCGGGCTTGGGACAGGGGAGGATACCAGGGCCACCTTCCCCAACCCAGGCCG
CGGGGGCCCGGCCTCCCCGATGCAGACCACAGCGCCCTCACGGGCTGCCCTCAGGCCGCGCAGCG
GGCAGCCGCCAGCCGTCACCCCGGGGAGCGTCCGTGGGGTGCCCAGGCACCCCACCCCGGCCCGG
GGCGCTCAGACGGCAGCAGACTGCTGGGCGGCGCGGGGACTACTTTCCACCGCCCCCTCGCGCCC
CGCCCCTTGTCCTCGCGCGGCGGAACGCTCCGCGCTGCGCCGGTGGCGGC

h-β-catenin-pro-mu4：

TCAGTAGGGATTAAAAATCATAATTCTAAATTTAAAGTATTTAAATTATAGAAAAATGATTCCAT

TATTTTGGTAATGGAACAGAGAAATACAAATATAGAAACGTTTTTATCCTTTAGATCTTCTAATG
GTGCAGATATTTCTTAACCAATTTCAAGAGTGCCTTATTCAAAAACAAAAAACCTGCACATAGAA
AAAGCAAACATGTTTTTTTAAAAAAAGCCCTGTAAAAGTGGTTAATCTGTCGTCTGATTTTCAAT
GTATGACTTTAATCAATTGCATATTCCTTAATTCTGCAAATGACAATTAAAATTAGGAATCAGTA
CCTGAAAACGCCATTTATACTTTTGAGTTTATATTAGAAACACGGTTTTGATGAAATACCTTTTT
CGGTTCAATCTTTTTGAATTGTGACCACAACCAATAGCTACACTTAGATTCTACAGAATTAGTAG
GAAAGATACAAATGAGAAGGTCTTTCTAGACAATGGATCTCACTGAACATCATCTTAGTGTGTAG
ACTTTTTCCACTGCAGTGGCCCACATGTGCTATGGTGACAGACGGCAGTTGGCATTACCACTTAT
ATAGTAAAATATCTTTAAGACAAAAATAAAAGTTATTTAAAGAATACGCTGGCCCTGAAACATGA
GCTGTGCCTTAAACTACATATCCTACCTCCATGAGTGAATGAATAAGTGGGTTTTCCGCCTGCAT
CTATGTGTTAAGACCTATTGTTTGCTTGTATTAATTGTAGTCCTGGGATAAAAGCACAGAGGTAA
CTTTCACTGCTGCTTTTTGTACTCTCTCCAATGTTTTGGAGGAAAAATAAGCACAAACAATAGCC
TAGAGAAACTGAATCGATCATACTTGTTGCAGCTTCGACAAACGTCAATTTTGCTGCATTAGAAT
GGGAAACATTTTTCAGTCTATTGAAATGAATTACAAACGTTTTTAAATAGAAAATTAGTTAAAAA
ATTGGAGGCTGCTTAATCGATAGCTTTCTCTATAAACATACTTGGATTTCACAAATAAGTAATAC
CGTAAAAATCTTCTTCTCCAAAGAAAAATCCCCACAAATAAATCTATTGATACCTAGTGACAAGT
GGAACCAGATAAAAATGGAATCTATAAGAATTAACCTAATTGACAGCGCTCTGGAGCTAATCCAT
TTCCATTAGgTcGtGgGtAcCAGTAGGTACTCCTAAGGACTTGTTGAATTGCGGCTTGGCGCCC
GTTCTACGGAGAGTTCACAGCCTTCGTGAGTGGGGACAGAAGGCGGCTCGGCCCGGTGATTCAGG
TCGAAATTCAAGCTGAACAGCCTGCTGAGAGGTGGGATCCACCATCCGGACAGTGGGGGGCTTTG
GGGGTGCTGTGAGACTGGGCTGCGACCCAGGTCCAGCAGGGAGTGTGCGGCACAGACCACAAGGT
CGGCGAGGCCCCCTAACCCGCGCCCGGCCGGGAACCCGCAGACCAGCGACGGGGCAGCTGCGGGG
CCAGGAGCGCCCCAAGACGGGCGGGCGCTGAACCCGAGCCCCTGCCGCCGCCCTGGCCCCGAACT
TCCGCCCTCCCAGGACCTGTCCCGGCCGCCCCGAGCGGTACTCGAAGGCCGGGGCCGAGATGCCA
CCTTCCGCAGGCCGCGGGAAAGGCGCGCCGAGTCCTGCAGCTGCTCTCCCGGTTCGGGAAACGCG
CGGGGCGGGGGCGTCGGGCTTGGGACAGGGGAGGATACCAGGGCCACCTTCCCCAACCCAGGCCG
CGGGGGCCCGGCCTCCCCGATGCAGACCACAGCGCCCTCACGGGCTGCCCTCAGGCCGCGCAGCG
GGCAGCCGCCAGCCGTCACCCCGGGGAGCGTCCGTGGGGTGCCCAGGCACCCCACCCCGGCCCGG
GGCGCTCAGACGGCAGCAGACTGCTGGGCGGCGCGGGGACTACTTTCCACCGCCCCCTCGCGCCC
CGCCCCTTGTCCTCGCGCGGCGGAACGCTCCGCGCTGCGCCGGTGGCGGC

3. The pcDNA3.1 vector map is as follows:

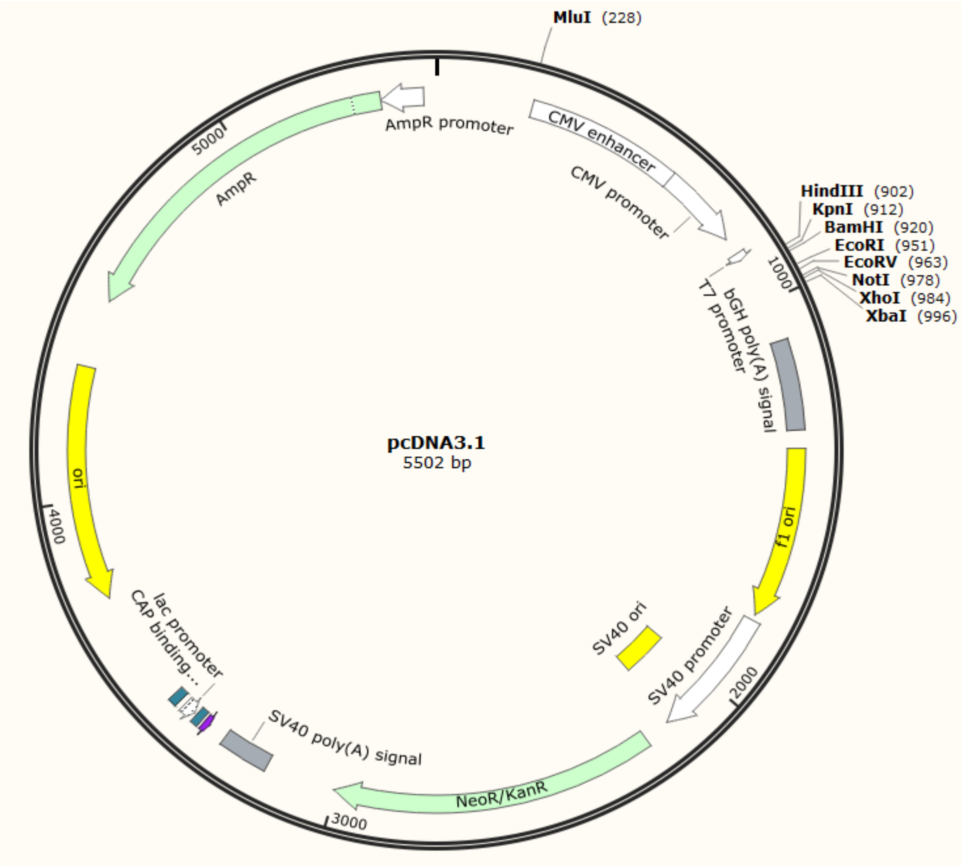

4. Human-FoxC1 sequence information

ATGCAGGCGCGCTACTCCGTGTCCAGCCCCAACTCCCTGGGAGTGGTGCCCTACCTCGGCGGCGA
GCAGAGCTACTACCGCGCGGCGGCCGCGGCGGCCGGGGGCGGCTACACCGCCATGCCGGCCCCCA
TGAGCGTGTACTCGCACCCTGCGCACGCCGAGCAGTACCCGGGCGGCATGGCCCGCGCCTACGGG
CCCTACACGCCGCAGCCGCAGCCCAAGGACATGGTGAAGCCGCCCTATAGCTACATCGCGCTCAT
CACCATGGCCATCCAGAACGCCCCGGACAAGAAGATCACCCTGAACGGCATCTACCAGTTCATCA
TGGACCGCTTCCCCTTCTACCGGGACAACAAGCAGGGCTGGCAGAACAGCATCCGCCACAACCTC
TCGCTCAACGAGTGCTTCGTCAAGGTGCCGCGCGACGACAAGAAGCCGGGCAAGGGCAGCTACTG
GACGCTGGACCCGGACTCCTACAACATGTTCGAGAACGGCAGCTTCCTGCGGCGGCGGCGGCGCT
TCAAGAAGAAGGACGCGGTGAAGGACAAGGAGGAGAAGGACAGGCTGCACCTCAAGGAGCCGCCC
CCGCCCGGCCGCCAGCCCCCGCCCGCGCCGCCGGAGCAGGCCGACGGCAACGCGCCCGGTCCGCA
GCCGCCGCCCGTGCGCATCCAGGACATCAAGACCGAGAACGGTACGTGCCCCTCGCCGCCCCAGC
CCCTGTCCCCGGCCGCCGCCCTGGGCAGCGGCAGCGCCGCCGCGGTGCCCAAGATCGAGAGCCCC
GACAGCAGCAGCAGCAGCCTGTCCAGCGGGAGCAGCCCCCCGGGCAGCCTGCCGTCGGCGCGGCC
GCTCAGCCTGGACGGTGCGGATTCCGCGCCGCCGCCGCCCGCGCCCTCCGCCCCGCCGCCGCACC
ATAGCCAGGGCTTCAGCGTGGACAACATCATGACGTCGCTGCGGGGGTCGCCGCAGAGCGCGGCC
GCGGAGCTCAGCTCCGGCCTTCTGGCCTCGGCGGCCGCGTCCTCGCGCGCGGGGATCGCACCCCC
GCTGGCGCTCGGCGCCTACTCGCCCGGCCAGAGCTCCCTCTACAGCTCCCCCTGCAGCCAGACCT
CCAGCGCGGGCAGCTCGGGCGGCGGCGGCGGCGGCGCGGGGGCCGCGGGGGGCGCGGGCGGCGCC
GGGACCTACCACTGCAACCTGCAAGCCATGAGCCTGTACGCGGCCGGCGAGCGCGGGGGCCACTT
GCAGGGCGCGCCCGGGGGCGCGGGCGGCTCGGCCGTGGACGACCCCCTGCCCGACTACTCTCTGC
CTCCGGTCACCAGCAGCAGCTCGTCGTCCCTGAGTCACGGCGGCGGCGGCGGCGGCGGCGGGGGA

GGCCAGGAGGCCGGCCACCACCCTGCGGCCCACCAAGGCCGCCTCACCTCGTGGTACCTGAACCA
GGCGGGCGGAGACCTGGGCCACTTGGCGAGCGCGGCGGCGGCGGCGGCCGCAGGCTACCCGG
GCCAGCAGCAGAACTTCCACTCGGTGCGGGAGATGTTCGAGTCACAGAGGATCGGCTTGAACAAC
TCTCCAGTGAACGGGAATAGTAGCTGTCAAATGGCCTTCCCTTCCAGCCAGTCTCTGTACCGCAC
GTCCGGAGCTTTCGTCTACGACTGTAGCAAGTTTTGA
